# Supplementary material for: Mapping single‐cell responses to population‐level dynamics during antibiotic treatment
Source: Mol Syst Biol. 2023 May 10;19(7):e11475. doi: 10.15252/msb.202211475 (PMC10333910; doi:10.15252/msb.202211475)
Supplement: Supplementary file 1 — Appendix [file MSB-19-e11475-s004.pdf]

Appendix

Mapping single-cell responses to population-level dynamics during antibiotic treatment

Kyeri Kim[1,2], Teng Wang[1,2], Helena R. Ma[1,2], Emrah Şimşek[1,2], Boyan Li[3], Virgile Andreani[4,5], and Lingchong You[1,2,6,7]

[1]Department of Biomedical Engineering, Duke University, USA

[2]Center for Quantitative Biodesign, Duke University, USA

[3]Integrated Science Program, Yuanpei College, Peking University, China

[4]Biomedical Engineering Department, Boston University, USA

[5]Biological Design Center, Boston University, USA

[6]Center for Genomic and Computational Biology, Duke University, USA

[7]Department of Molecular Genetics and Microbiology, Duke University School of Medicine, USA

*Correspondence and requests for materials should be addressed to Lingchong You.

E-mail: you@duke.edu; Tel: 919-660-8408; Fax: 919-668-0795

Table of contents

Appendix Table S1 ———— page 3

Appendix Figures S1 – S9 ———— page 4 – 15

Appendix Supplementary Methods

- *Mathematical model of damage accumulation* ———— page 16 – 18
- *Derivation of the hazard function formula* ———— page 18 – 19

Appendix Reference ———— page 20

Strain	Antibiotics	Dose (μg/ml)	Temp. (°C)	nCells	L_c (μm)	H	R-square
MG1655	Carbenicillin	20	27	110	22.97	5.00	0.9980
MG1655	Carbenicillin	20	37	110	22.33	4.45	0.9948
MG1655	Carbenicillin	50	27	104	10.96	3.96	0.9992
MG1655	Carbenicillin	50	37	110	10.99	2.69	0.9961
MG1655	Carbenicillin	100	27	110	7.25	2.07	0.9914
MG1655	Carbenicillin	100	37	106	6.97	3.72	0.9984
MG1655	Cefotaxime	20	27	100	6.30	5.76	0.9993
MG1655	Cefotaxime	20	37	100	6.37	7.08	0.9998
MG1655	Cefotaxime	100	27	100	4.03	8.15	0.9999
MG1655	Cefotaxime	100	37	100	4.47	7.55	0.9999
MG1655	Amoxicillin	6.25	27	100	11.36	4.08	0.9977
MG1655	Amoxicillin	6.25	37	100	9.12	2.97	0.9946
MG1655	Amoxicillin	25	27	100	5.16	10.44	1.0000
MG1655	Amoxicillin	25	37	110	4.85	11.38	1.0000
ESBL053	Amoxicillin	6.25	37	100	7.46	7.80	0.9997
ESBL053	Amoxicillin	25	37	100	5.51	8.04	0.9999
ESBL055	Amoxicillin	6.25	37	100	8.74	9.23	0.9997
ESBL055	Amoxicillin	25	37	100	6.75	10.48	0.9999
ESBL058	Amoxicillin	6.25	37	100	7.16	7.36	0.9998
ESBL058	Amoxicillin	25	37	100	5.04	8.39	0.9999

Appendix Table S1. Fitted results of P_L in antibiotic dose and temperature modulation assays.

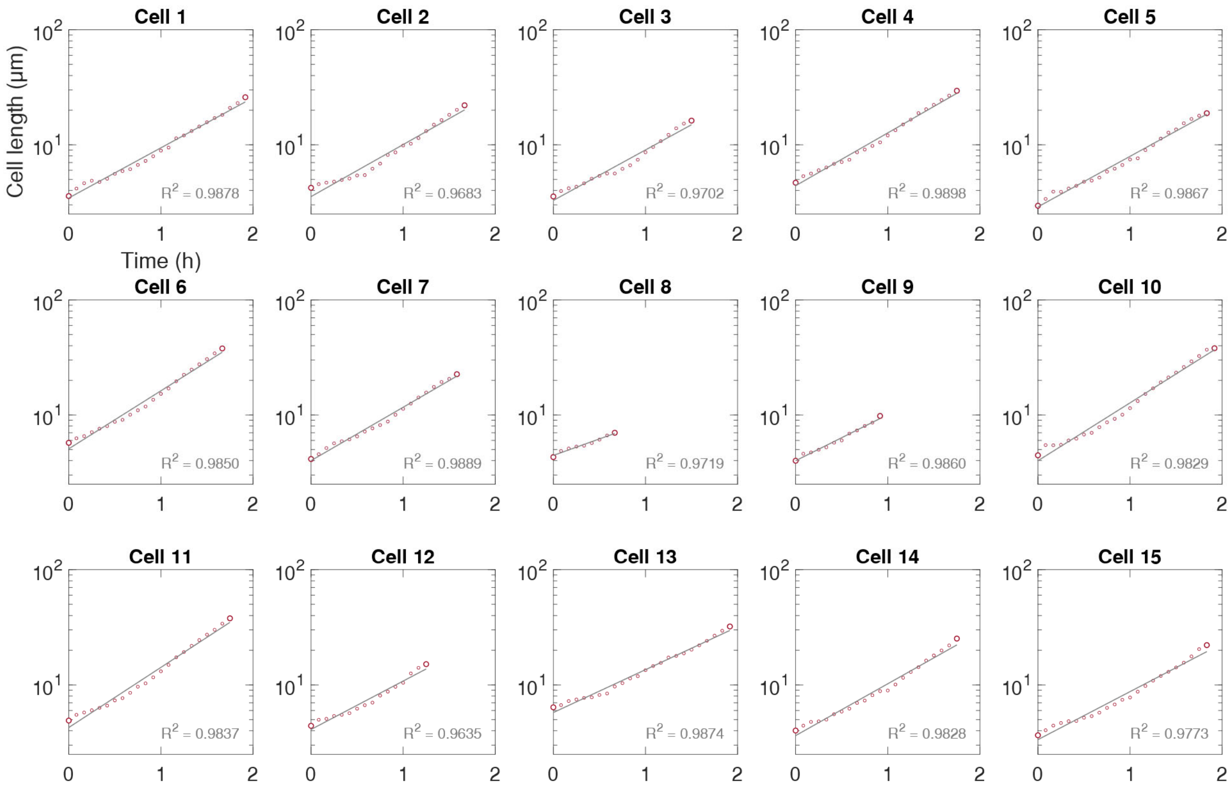

Appendix Figure S1. Intermediate cell lengths of beta-lactam antibiotic-induced filamentation.

The long-axis lengths of 15 additional cells were tracked as the one in **Figure 1B**. The initial and final lengths were marked in larger circles than the markers of intermediate lengths. Linear regression results on log-scaled cell length over time were shown in a solid gray line, supporting that cells elongate exponentially until burst.

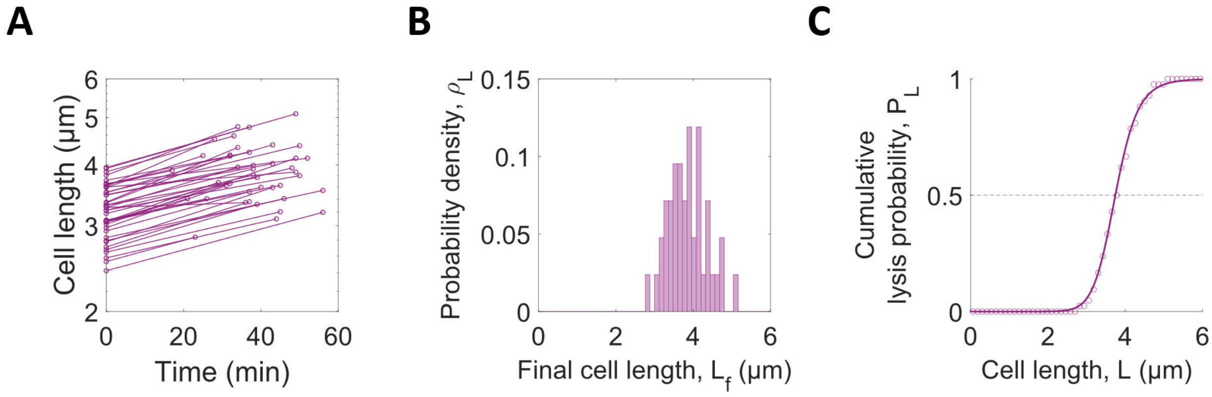

Appendix Figure S2. D-cycloserine-induced lysis probability in a single cell increased with the extent of filamentation.

A. Initial and final lengths of cells over time. From the raw data, only cells that elongated and lysed after treatment were chosen for analysis. Line plots show log scaled initial and final length over time.

B. Probability density distribution from final length. Probability density distribution (ρ_L) over the final length was taken by normalizing probability distributions.

C. Cumulative lysis probability increases with elongation during treatment. Cumulative probabilities (P_L) of the ρ_L are shown in dot plot. Solid line shows the log-logistic fitted P_L.

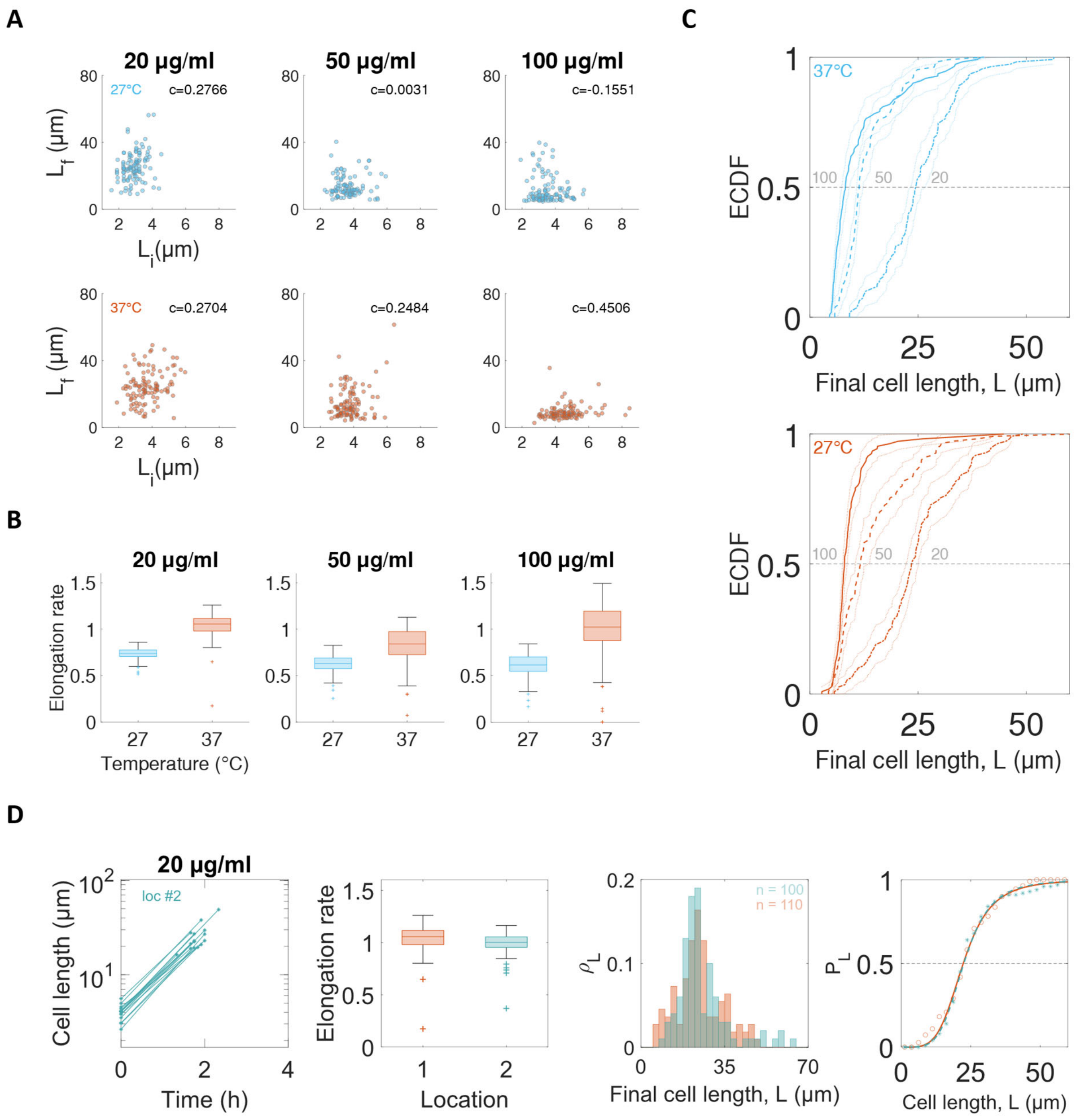

Appendix Figure S3. Effects of initial cell length and incubation temperature on carbenicillin-induced elongation.

A. Final length is independent from initial length. Scatter plots of final length over initial length show weak correlations between initial and final lengths in all conditions. c is the correlation coefficient as a measure of linear independence and printed on the top right of each panel.

B. Temperature modulates elongation rate. Individual elongation rates were calculated with initial and final length measurements of individual cells. In accordance with the slopes in **Figure 2A**, elongation rate was higher at 37 °C vs. 27 °C.

C. Confidence intervals of empirical cumulative distribution functions (ECDF). ECDF of the final length distributions were plotted in thicker lines, while 95% confidence intervals were plotted in thinner dotted lines using the MATLAB 'ecdf' function with 'Bound' option. Carbenicillin concentrations (μg/ml) were marked near the distributions.

D. $\boldsymbol{P_L}$ was highly consistent in two imaging locations of one sample. P_L of cells that were located in the second imaging locations of the same sample (**Figure 2A**, 20μg/ml, 37°C) showed highly consistent fitted P_L ($L_c = 22.55$, and $H = 4.71$).

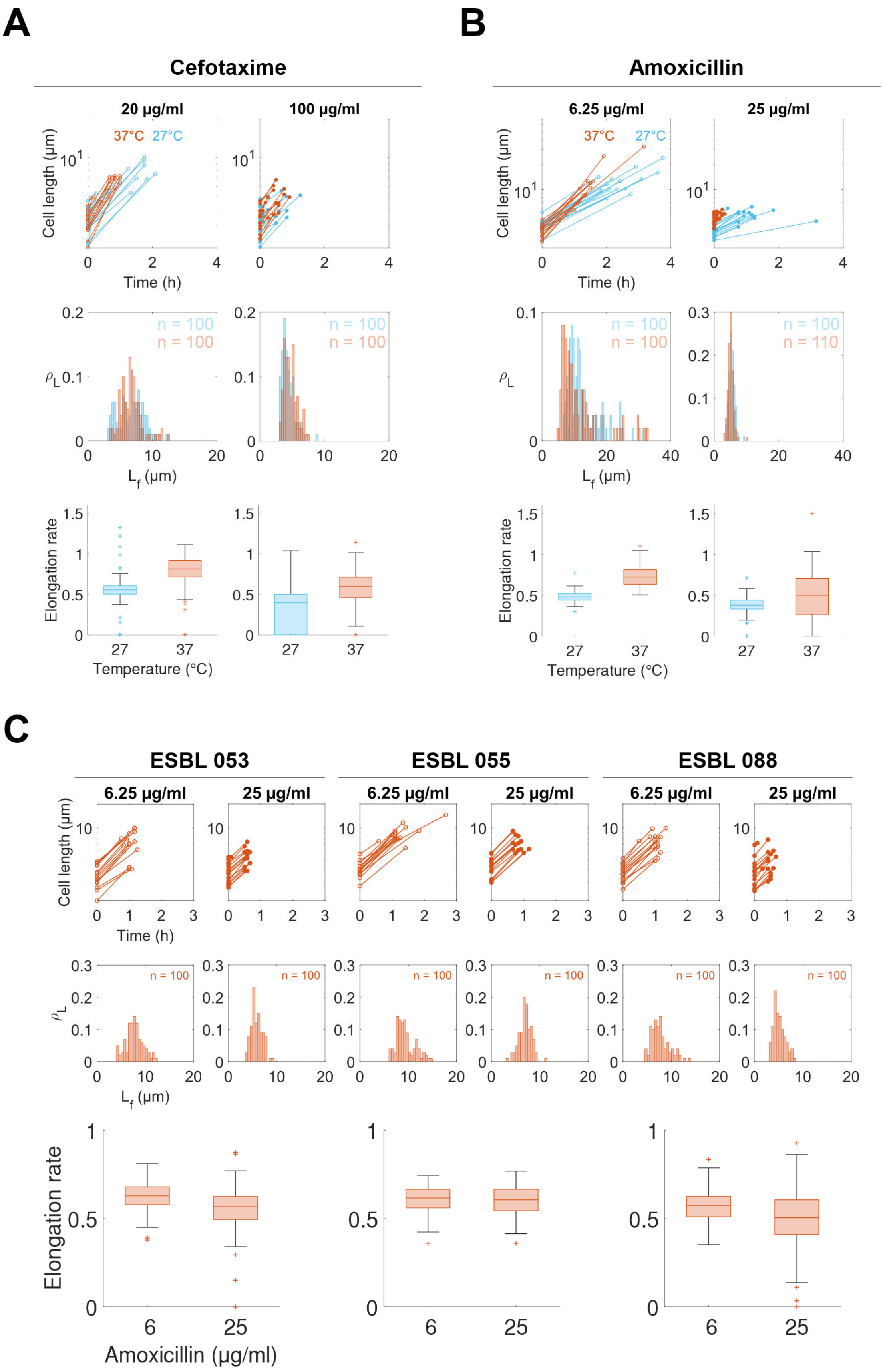
A
Cefotaxime
20 µg/ml
100 µg/ml
37°C 27°C
Cell length (µm)
Time (h)
n = 100
n = 100
ρ_L
L_f (µm)
Elongation rate
Temperature (°C)
B
Amoxicillin
6.25 µg/ml
25 µg/ml
n = 110
C
ESBL 053
ESBL 055
ESBL 088
Amoxicillin (µg/ml)

Appendix Figure S4. Final length measurements for finding P_L in other beta-lactams and ESBL-producing strains.

A-B. Cefotaxime and amoxicillin treatment in *E. coli* MG1655 with temperature modulation. Two doses of cefotaxime (20 and 100 μg/ml) and amoxicillin (6.25 and 25 μg/ml) at two temperatures (27°C in blue and 37°C in red) were tested. Initial and final lengths of the first 15 cells are shown in the first row for display. Lysis probability densities from the final lengths of all measured cells are shown in the second row. Elongation rates of all measured cells are shown in the third row.

C. Amoxicillin-treated ESBL-producing *E. coli* from the patient isolate library with Bla inhibition. Clavulanate acid (50 μg/ml) was added with amoxicillin (6.25 and 25 μg/ml) to the clinical isolates and incubated at 37 °C. Cell length, probability density, and elongation rate plots are derived by the same methods in panels **A** and **B**. At the later stage of low-dose amoxicillin treatment, these cells formed localized swelling (Burdett & Murray, 1974; Cushnie *et al*, 2016)—a gradually inflated body from the middle of its cylindrical region—and the lab strain formed a bulb through the cell wall without changing the cell width. Therefore, we note that the final length of amoxicillin treated (6.25 μg/ml) cells could be slightly longer than the measured length, because the biomass that contributed to extending the width may have extended length if cells did not form local swell.

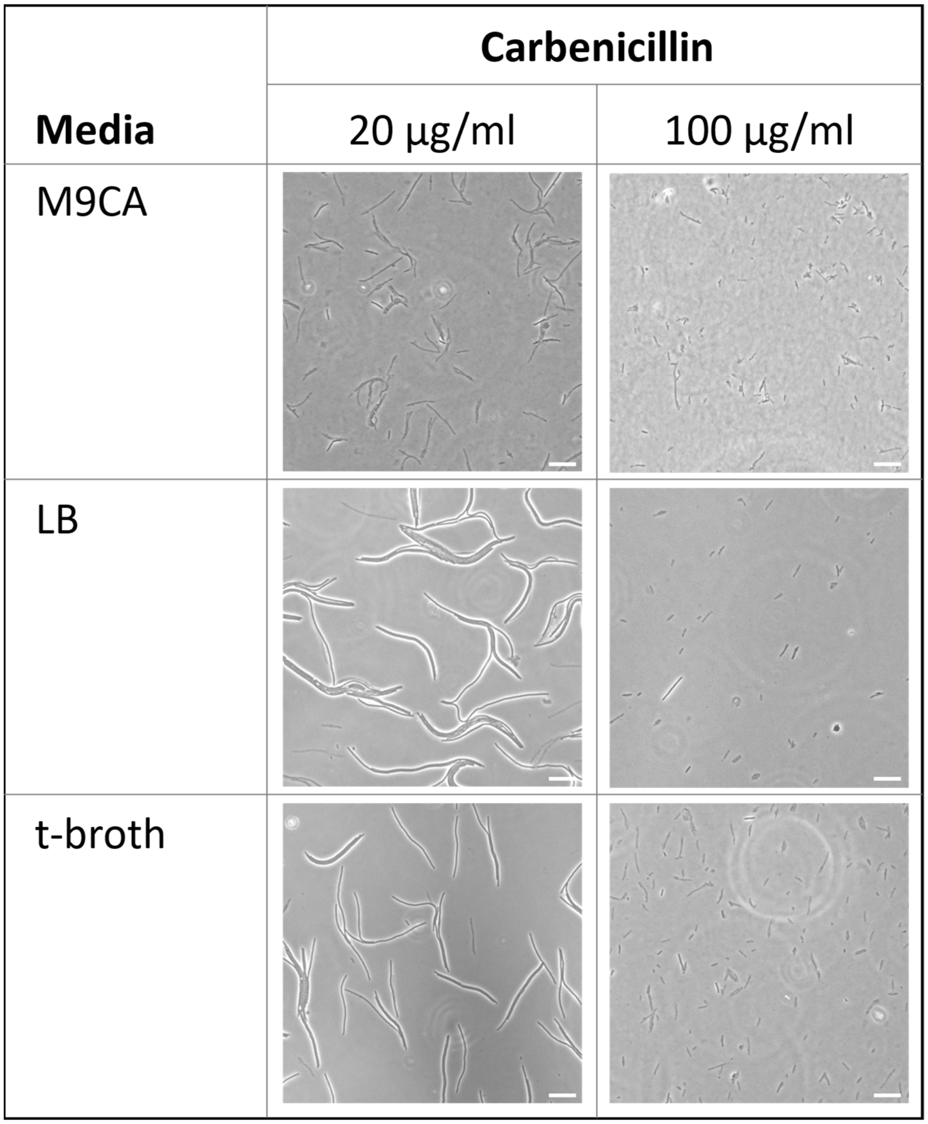

Appendix Figure S5. Dose-dependency of L_c maintained in different growth media.

Exponentially growing cells were loaded on different media gel with the same absolute carbenicillin doses for time-lapse microscopy. Images taken after around 6 to 8 hours of antibiotic exposure were picked for displaying low-dose carbenicillin-treated cells. The inverse relationship of L_c and carbenicillin dose was conserved in each medium. Compared to cells grown in M9CA medium, used in **Figure 2** experiments, the lengths of the cells grown in LB and t-broth were longer; therefore, L_c is sensitive to growth media. Scale bars: 20μm.

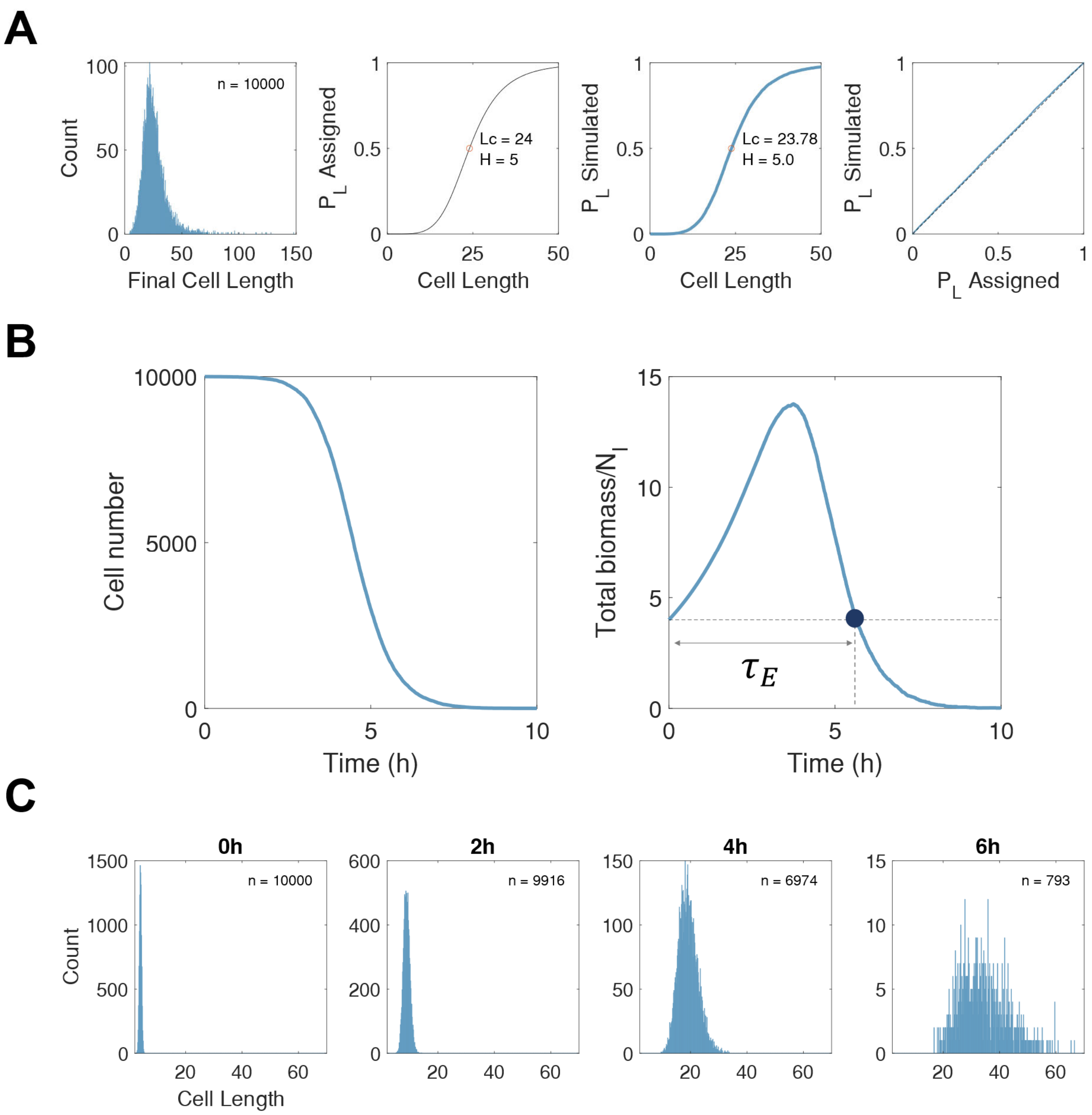

Appendix Figure S6. Stochastic simulation confirms the single-cell level lysis profiles.

A. Stochastic final length simulations. Stochastic simulation of final length distribution (the first panel) of a population with 10,000 cells was done with an assigned cumulative probability $P_A = \frac{L^H}{L^H + Lc^H}$ (L_c=24 and H=5, the second panel). The cumulative distribution function from the distribution, simulated P_L (the third panel), was achieved and compared to the assigned P_L (the last panel).

B. Simulated population responses via collection of individual responses. Simulation results show that the cell number only decreases because the cells cannot divide but are lysed (left panel). However, the sum of the biomass of survivors (total biomass) increases due to elongation and low lysis probability during early exposure and then decreases due to higher lysis probability (right panel). The time point at which total biomass crosses the initial biomass is the effective elongation duration, τ_E.

C. Time-course cell length distributions. Survivor length distributions at 0 to 6h elongation.

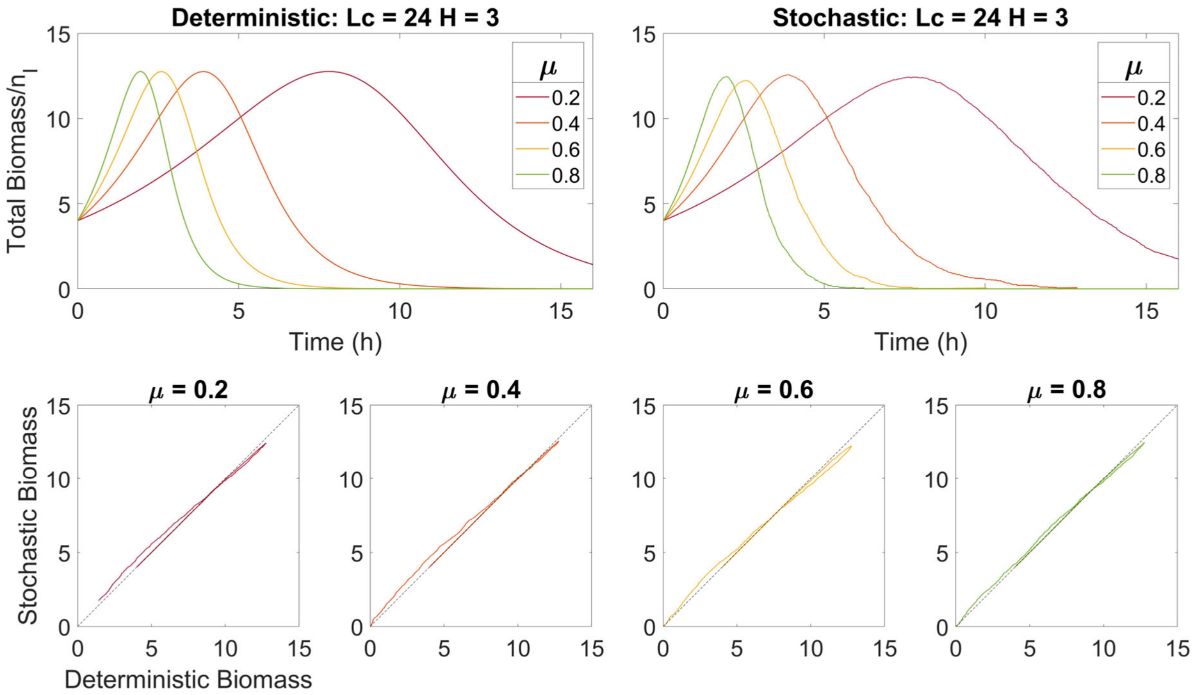

Appendix Figure S7. Deterministic models can represent populational biomass dynamics using average parameters of stochastic models.

Total biomass over time is shown in panel **A**, using the same average initial length and P_L parameters (L_c and H) with four different average elongation rates. Deterministic and stochastic simulations were compared 1:1 by using total biomass at every time step in panel **B**.

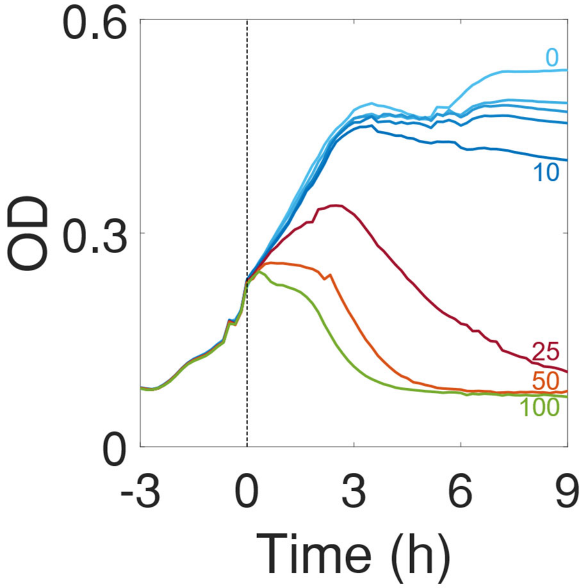

Appendix Figure S8. Dose-response population growth and lysis dynamics.

Carbenicillin (0, 1, 2.5, 5, 10, 25, 50, and 100 μg/ml) was added at time 0. Populations with low-dose antibiotics (≤ 10 μg/ml) show similar OD curves.

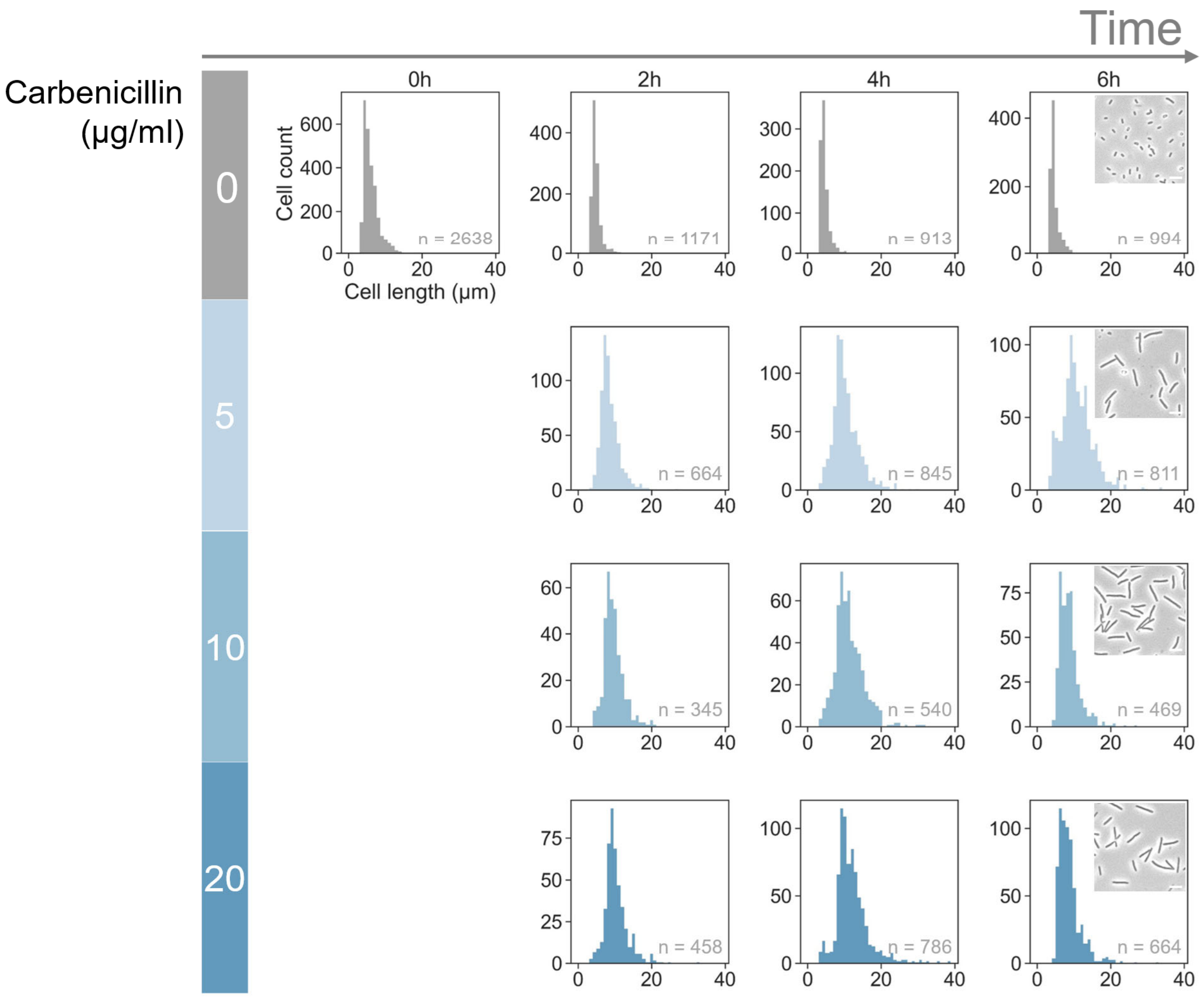

Appendix Figure S9. Cell length distributions following carbenicillin treatment over time.

Cell lengths were measured from the microscope images of sampled cells in time-course and dose-response experiments. The single-cell images were captured every 2 hours from the carbenicillin (0, 5, 10, and 20 μg/ml) addition at time 0. Long-axis length distributions were generated with the same number of bins. Scale bars: 10μm.

Appendix Supplementary Methods

Mathematical model of damage accumulation

We assumed that 1) reaching a threshold number (α) of unsuccessful peptidoglycan cross-linkages on the cell wall causes a loss of cell wall integrity and triggers lysis and 2) the number of failures being made per unit cell length is the damage rate, β([A]), which increases with antibiotic dose. According to our model, final length is then the length of cells when α failures have been acquired: its density distribution follows a gamma distribution of which the cumulative distribution is also a sigmoid.

We define the probability of having one cross-linking failure per unit length as damage rate (β) and allow only one failure in unit length. That is, for any $\Delta L \to 0$, the probability of one damage is $p_1(\Delta L) = \beta \Delta L + o(\Delta L)$, no damage is is $p_0(\Delta L) = 1 - \beta \Delta L$, and multiple damages is $p_{n \geq 2}(\Delta L) = 0$. Since we assume that the cross-linkage failure accumulates independently, an infinitesimal elongation (dL) of L-length cell without damage should follow:

$$p_0(L + dL) = p_0(L)p_0(dL) = p_0(L)(1 - \beta dL). \qquad (S1)$$

Therefore,

$$\frac{dp_0(L)}{dL} = -\beta p_0(L). \qquad (S2)$$

Similarly, the probability of an infinitesimal elongation (dL) of L-length cell with n damages should follow:

$$p_n(L + dL) = p_{n-1}(L)p_1(dL) + p_n(L)p_0(dL) = p_{n-1}(L)\beta dL + p_n(L)(1 - \beta dL), \qquad (S3)$$

$$\frac{dp_n(L)}{dL} + \beta p_n(L) = \beta p_{n-1}(L), \qquad (S4)$$

$$\frac{d}{dL}\left[e^{\beta L} p_n(L)\right] = \beta e^{\beta L} p_{n-1}(L). \qquad (S5)$$

where Eq. S5 is a general recursive equation for series $\{p_0(L), p_1(L), p_2(L), \cdots\}$. Combining Eq. S2 and $p_0(0) = 0$, we have $p_0(L) = e^{-\beta L}$. Then, the probability of L-length cell with n damages is:

$$p_n(L) = \frac{(\beta L)^n}{n!} e^{-\beta L}. \tag{S6}$$

If we assume that the lysis occurs when a cell attains α damages, the final length distribution of cells with α damages ($P_L(\alpha)$) should follow:

$$P_L(\alpha) = 1 - \sum_{i=0}^{\alpha-1} p_i(L) = 1 - e^{-\beta L} \sum_{i=0}^{\alpha-1} \frac{(\beta L)^i}{i!}. \tag{S7}$$

Here, we note that Eq. S7 is the cumulative density function of final lengths and known as the Gamma distribution. Essentially, the model introduces the Poisson process on damage accumulation and the length to the α-th damage follows a Gamma distribution.

In accordance with the log-logistic fitting, the dependence of P_L on L in gamma distributions shifts leftward at a larger β with constant α (**Figure EV1A**). This trend is consistent with the experimental observations: all else being equal, a higher antibiotic dose (indicates larger β) resulted in a smaller L_c (the length at the mean of cumulative distributions) (**Figure 2**).

Our numerical analysis showed that L_c was proportional to α/β, and H increased approximately with the square root of α (**Figure EV1B**). These correlations emerge from the analytic solutions under the limiting case when $H \gg 1$.

Briefly, we assume the log-logistic distribution (with its CDF being the Eq 1 from direct data fitting) and the gamma distribution (arising from the damage-accumulation model) approximately describe the same data. Thus, the means and the variances of two-fitted distributions should be equal to each other:

$$\frac{L_c \frac{\pi}{H}}{sin \frac{\pi}{H}} = \frac{\alpha}{\beta}, \tag{S8}$$

$$L_c{}^2\left(\frac{\frac{2\pi}{H}}{sin\frac{2\pi}{H}}-\frac{\left(\frac{\pi}{H}\right)^2}{sin^2\frac{\pi}{H}}\right)=\frac{\alpha}{\beta^2}. \tag{S9}$$

Further simplification of Eq. S8 shows that $L_c \approx \frac{\alpha}{\beta}$ since $\frac{\frac{\pi}{H}}{sin\frac{\pi}{H}} \approx 1$ when $H \gg 1$.

Plugging $L_c \approx \frac{\alpha}{\beta}$ into Eq. S9 gives:

$$\frac{\frac{2\pi}{H}}{sin\frac{2\pi}{H}}-\frac{\left(\frac{\pi}{H}\right)^2}{sin^2\frac{\pi}{H}}=\frac{1}{\alpha}. \tag{S10}$$

Expanding the left-hand-side terms to the second order using $\frac{x}{sinx} = 1 + \frac{1}{6}x^2 + O(x^4)$ gives:

$$1+\frac{1}{6}\left(\frac{2\pi}{H}\right)^2-\left(1+\frac{1}{6}\left(\frac{2\pi}{H}\right)^2\right)^2=\frac{1}{\alpha}. \tag{S11}$$

Further simplification of the left-hand-side terms to the second order of H derives $H \approx \pi\sqrt{\frac{\alpha}{3}}$. These results indicate that measured L_c and H are approximately determined by the maximum number of accumulated cross-linkage failures (α) and the damage rate (β) to retain the cell wall integrity.

Derivation of the hazard function formula

The hazard function, also known as the instantaneous death rate or instantaneous failure rate, was initially introduced by Gompertz in 1825 (Gompertz). It is often used in modeling the death of individuals in an age-distributed population, where the independent variable is time (age). However, it can be defined in terms of any monotonically increasing variable.

The Eq. 2, $P_H(L) = \frac{\rho_L}{1-P_L}$, is the hazard function formula, expressed as a function of cell length (L), where length is monotonically increasing variable between initial to final lengths. Below is a derivation of the hazard function formula from its definition (Collett, 1994).

In our study, we define $P_H(L)$ in terms of cell length (L):

$$P_H(L) \equiv \lim_{dL\to 0} \frac{P(L < l < L + dL | l > L)}{dL} \ . \qquad (S12)$$

By definition, the hazard function describes the instantaneous lysis rate of an individual cell at cell length L, that has survived to L. The numerator of Eq. S12 is a conditional probability, which can be expanded and rearranged according to the Bayes theorem:

$$\begin{aligned} P(L < l < L + dL | l > L) &= \frac{P(l > L | L < l < L + dL) P(L < l < L + dL)}{P(l > L)} \\ &= \frac{P(L < l < L + dL)}{P(l > L)}, \end{aligned} \qquad (S13)$$

as $P(l > L | L < l < L + dL)$ is always 1, reflecting certainty.

The denominator of Eq. S13 is the survival function of the population:

$$P(l > L) = 1 - cumulative\ lysis\ probability = 1 - P_L. \qquad (S14)$$

Plugging Eqs. S13 and S14 into Eq. S12, we have:

$$\begin{aligned} P_H(L) &= \lim_{dL\to 0} \frac{P(L < l < L + dL)}{P(l > L)} \frac{1}{dL} \\ &= \lim_{dL\to 0} \frac{P(l < L + dL) - P(l < L)}{dL} \frac{1}{P(l > L)} \\ &= \frac{1}{P(l > L)} \lim_{dL\to 0} \frac{P(l < L + dL) - P(l < L)}{dL} \\ &= \frac{1}{1 - P_L} \frac{dP_L}{dL} \\ &= \frac{\rho_L}{1 - P_L}, \end{aligned}$$

which is the hazard function formula.

Appendix Reference

Burdett ID, Murray RG (1974) Septum formation in Escherichia coli: characterization of septal structure and the effects of antibiotics on cell division. *Journal of bacteriology* 119: 303-324
Collett D (1994) *Modelling Survival Data in Medical Research*. Springer US
Cushnie TPT, O'Driscoll NH, Lamb AJ (2016) Morphological and ultrastructural changes in bacterial cells as an indicator of antibacterial mechanism of action. *Cellular and Molecular Life Sciences* 73: 4471-4492
Gompertz BP XXIV. On the nature of the function expressive of the law of human mortality, and on a new mode of determining the value of life contingencies. In a letter to Francis Baily, Esq. F. R. S. &c. *Philosophical Transactions of the Royal Society of London*: 513 - 583
