# Supplementary material for: Mapping single‐cell responses to population‐level dynamics during antibiotic treatment
Source: Mol Syst Biol. 2023 May 10;19(7):e11475. doi: 10.15252/msb.202211475 (PMC10333910; doi:10.15252/msb.202211475)
Supplement: Supplementary file 2 — Expanded View Figures PDF [file MSB-19-e11475-s001.pdf]

Expanded View Figures

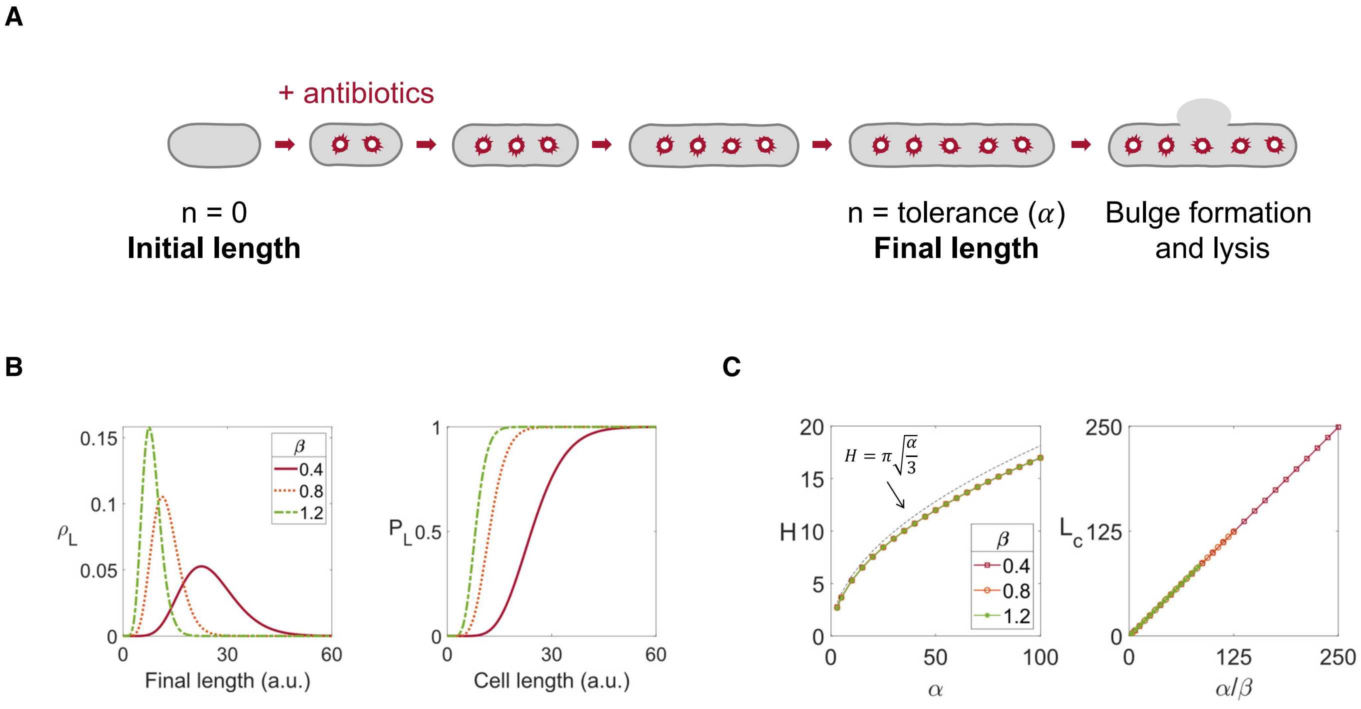

Figure EV1. Damage accumulation model provides a plausible interpretation of the log-logistic distribution through gamma distribution.

A Damage accumulates on the cell wall until lysed. A schematic diagram of the damage accumulation model for cell lysis. Upon addition of antibiotics, a cell is assumed to accumulate damage on its cell wall during elongation, with an antibiotic dose-dependent rate of β. When the total number of damages to a cell reaches a threshold value α, the cell formed a bulge and lysed in a short time.

B Final length follows a gamma distribution. Under the damage accumulation model, the final length of a cell that has α damages follows a gamma distribution with the parameters of α and β (see Appendix Supplementary Methods). Probability density function (PDF, ρ_L, left) and cumulative distribution function (CDF, P_L, right) of the three different rates ($\beta = 0.4$, 0.8, and 1.2) recapitulate the experimental distributions shown in Fig 2B. All three plots used a constant threshold ($\alpha = 10$).

C Parameters of gamma and log-logistic distributions are correlated. Gamma distributions that were generated with different parameter sets of α and β were fitted to log-logistic distributions ($P_L = \frac{L^H}{L^H + L_c^H}$). H was not sensitive to β (left) but highly sensitive to α (bottom left). Black dashed line shows the approximation of H to α in 2nd order. L_C was proportional to α/β (right). R^2 of all log-logistic fits were larger than 0.997.

 © 2023 The Authors
