# Supplementary material for: Mapping single‐cell responses to population‐level dynamics during antibiotic treatment
Source: Mol Syst Biol. 2023 May 10;19(7):e11475. doi: 10.15252/msb.202211475 (PMC10333910; doi:10.15252/msb.202211475)
Supplement: Supplementary file 4 [file MSB-19-e11475-s003.pdf]

molecular systems biology

Mapping single-cell responses to population-level dynamics during antibiotic treatment

Kyeri Kim, Teng Wang, Helena Ma, Emrah Şimşek, Boyan Li, Virgile Andreani, and Lingchong You
DOI: 10.15252/msb.202211475

Corresponding author(s): Lingchong You (you@duke.edu)

Review Timeline:

Submission Date:	23rd Nov 22
Editorial Decision:	9th Jan 23
Revision Received:	9th Mar 23
Editorial Decision:	24th Apr 23
Revision Received:	24th Apr 23
Accepted:	25th Apr 23

Editor: Jingyi Hou / Maria Polychronidou

Transaction Report:

(Note: With the exception of the correction of typographical or spelling errors that could be a source of ambiguity, letters and reports are not edited. Depending on transfer agreements, referee reports obtained elsewhere may or may not be included in this compilation. Referee reports are anonymous unless the Referee chooses to sign their reports.)

9th Jan 2023

Manuscript Number: MSB-2022-11475
Title: Mapping single-cell responses to population-level dynamics during antibiotic treatment
Author: Kyeri Kim
Teng Wang
Helena Ma
Emrah Şimşek
Boyan Li
Virgile Andreani
Lingchong You

Dear Lingchong,

Thank you for submitting your work to Molecular Systems Biology. I apologize for the delay in sending you a decision on your manuscript, which was due to the holiday season and the late arrival of the reviewers' report. We have now received feedback from three reviewers who agreed to evaluate your manuscript. As you will see from the reports below, the reviewers acknowledge the potential interest of the study. They raise however a series of concerns, which we would ask you to address in a major revision.

Since the reviewers' recommendations are rather clear, there is no need to reiterate all the points listed below. All issues raised by the reviewers need to be satisfactorily addressed. As you may already know, our editorial policy allows in principle a single round of major revision and it is therefore essential to provide responses to the reviewers' comments that are as complete as possible. Please feel free to contact me in case you would like to discuss in further detail any of the issues raised by the reviewers.

On a more editorial level, we would ask you to address the following issues:

- Please provide a .docx formatted version of the manuscript text (including legends for main figures, EV figures and tables). Please make sure that the changes are highlighted to be clearly visible.

- Please provide individual production quality figure files as .eps, .tif, .jpg (one file per figure).

-Please provide a .docx formatted letter INCLUDING the reviewers' reports and your detailed point-by-point responses to their comments. As part of the EMBO Press transparent editorial process, the point-by-point response is part of the Review Process File (RPF), which will be published alongside your paper.

-Please note that all corresponding authors are required to supply an ORCID ID for their name upon submission of a revised manuscript.

-We replaced Supplementary Information with Expanded View (EV) Figures and Tables that are collapsible/expandable online (see examples in http://msb.embopress.org/content/11/6/812). A maximum of 5 EV Figures can be typeset. EV Figures should be cited as 'Figure EV1, Figure EV2" etc... in the text and their respective legends should be included in the main text after the legends of regular figures.

Additional Tables/Datasets should be labeled and referred to as Table EV1, Dataset EV1, etc. Legends have to be provided in a separate tab in case of .xls files. Alternatively, the legend can be supplied as a separate text file (README) and zipped together with the Table/Dataset file.

For the figures and tables that you do NOT wish to display as Expanded View figures, they should be bundled together with their legends in a single PDF file called *Appendix*, which should start with a short Table of Content. Each legend should be below the corresponding Figure/Table in the Appendix. Appendix figures and tables should be referred to in the main text as: "Appendix Figure S1, Appendix Figure S2, Appendix Table S1" etc. See detailed instructions regarding expanded view here: https://www.embopress.org/page/journal/17444292/authorguide#expandedview.

-Before submitting your revision, primary datasets (and computer code, where appropriate) produced in this study need to be deposited in an appropriate public database (seehttp://msb.embopress.org/authorguide - dataavailability https://www.embopress.org/page/journal/17444292/authorguide#dataavailability).

Please remember to provide a reviewer password if the datasets are not yet public.

The accession numbers and database should be listed in a formal "Data Availability" section (placed after Materials & Method) that follows the model below (see also https://www.embopress.org/page/journal/17444292/authorguide#dataavailability). Please note that the Data Availability Section is restricted to new primary data that are part of this study.

Data availability

The datasets (and computer code) produced in this study are available in the following databases:

- RNA-Seq data: Gene Expression Omnibus GSE46843 (https://www.ncbi.nlm.nih.gov/geo/query/acc.cgi?acc=GSE46843)
- [data type]: [name of the resource] [accession number/identifier/doi] ([URL or identifiers.org/DATABASE:ACCESSION])

*** Note - All links should resolve to a page where the data can be accessed. ***

-At EMBO Press we ask authors to provide source data for the main figures. Our source data coordinator will contact you to discuss which figure panels we would need source data for and will also provide you with helpful tips on how to upload and organize the files.

- Our journal encourages inclusion of *data citations in the reference list* to directly cite datasets that were re-used and obtained from public databases. Data citations in the article text are distinct from normal bibliographical citations and should directly link to the database records from which the data can be accessed. In the main text, data citations are formatted as follows: "Data ref: Smith et al, 2001". In the Reference list, data citations must be labeled with "[DATASET]". A data reference must provide the database name, accession number/identifiers and a resolvable link to the landing page from which the data can be accessed at the end of the reference. Further instructions are available at .

- We updated our journal's competing interests policy in January 2022 and request authors to consider both actual and perceived competing interests. Please review the policy https://www.embopress.org/competing-interests and update your competing interests if necessary.

Please use the heading "Disclosure statement and competing interests".

- All Materials and Methods need to be described in the main text. We would encourage you to use 'Structured Methods', our new Materials and Methods format. According to this format, the Material and Methods section should include a Reagents and Tools Table (listing key reagents, experimental models, software and relevant equipment and including their sources and relevant identifiers) followed by a Methods and Protocols section in which we encourage the authors to describe their methods using a step-by-step protocol format with bullet points, to facilitate the adoption of the methodologies across labs. More information on how to adhere to this format as well as downloadable templates (.doc or .xls) for the Reagents and Tools Table can be found in our author guidelines: < https://www.embopress.org/page/journal/17444292/authorguide#researcharticleguide>. An example of a Method paper with Structured Methods can be found here: .

-Regarding data quantification:
Please ensure to specify the name of the statistical test used to generate error bars and P values, the number (n) of independent experiments (please specify technical or biological replicates) underlying each data point and the test used to calculate p-values in each figure legend. Discussion of statistical methodology can be reported in the materials and methods section, but figure legends should contain a basic description of n, P and the test applied.
Graphs must include a description of the bars and the error bars (s.d., s.e.m.).
Please also include scale bars in all microscopy images.

- Please provide a "standfirst text" summarizing the study in one or two sentences (approximately 250 characters, including space), three to four "bullet points" highlighting the main findings and a "synopsis image" (550px width and 400-600 px height, PNG format) to highlight the paper on our homepage.
Here are a couple of examples:
https://www.embopress.org/doi/10.15252/msb.20199356
https://www.embopress.org/doi/10.15252/msb.20209475
https://www.embopress.org/doi/10.15252/msb.209495

When you resubmit your manuscript, please download our CHECKLIST (https://www.embopress.org/pb-assets/embo-site/EMBO%20Press%20Author%20Checklist-1642513524327.xlsx) and include the completed form in your submission. *Please note* that the Author Checklist will be published alongside the paper as part of the transparent process (https://www.embopress.org/page/journal/17444292/authorguide#transparentprocess).

If you feel you can satisfactorily deal with these points and those listed by the referees, you may wish to submit a revised version of your manuscript. Please attach a covering letter giving details of the way in which you have handled each of the points raised by the referees. A revised manuscript will be once again subject to review and you probably understand that we can give you no guarantee at this stage that the eventual outcome will be favorable.

I look forward to receiving your revised manuscript soon.

Sincerely,
Jingyi

Jingyi Hou, PhD
Scientific Editor
Molecular Systems Biology

We realize that it is difficult to revise to a specific deadline. In the interest of protecting the conceptual advance provided by the work, we recommend a revision within 3 months (9th Apr 2023). Please discuss the revision progress ahead of this time with the editor if you require more time to complete the revisions. Use the link below to submit your revision:

https://msb.msubmit.net/cgi-bin/main.plex

IMPORTANT: When you send your revision, we will require the following items:
1. the manuscript text in LaTeX, RTF or MS Word format
2. a letter with a detailed description of the changes made in response to the referees. Please specify clearly the exact places in the text (pages and paragraphs) where each change has been made in response to each specific comment given
3. three to four 'bullet points' highlighting the main findings of your study
4. a short 'blurb' text summarizing in two sentences the study (max. 250 characters)
5. a 'thumbnail image' (550px width and max 400px height, Illustrator, PowerPoint or jpeg format), which can be used as 'visual title' for the synopsis section of your paper.
6. Please include an author contributions statement after the Acknowledgements section (see https://www.embopress.org/page/journal/17444292/authorguide)
7. Please complete the CHECKLIST available at (https://bit.ly/EMBOPressAuthorChecklist).
Please note that the Author Checklist will be published alongside the paper as part of the transparent process (https://www.embopress.org/page/journal/17444292/authorguide#transparentprocess).
8. When assembling figures, please refer to our figure preparation guideline in order to ensure proper formatting and readability in print as well as on screen:
https://bit.ly/EMBOPressFigurePreparationGuideline
See also figure legend guidelines: https://www.embopress.org/page/journal/17444292/authorguide#figureformat
9. Please note that corresponding authors are required to supply an ORCID ID for their name upon submission of a revised manuscript (EMBO Press signed a joint statement to encourage ORCID adoption).
(https://www.embopress.org/page/journal/17444292/authorguide#editorialprocess)
Currently, our records indicate that the ORCID for your account is 0000-0003-3725-4007.

Please click the link below to modify this ORCID:
Link Not Available

10. At EMBO Press we ask authors to provide source data for the main manuscript figures. Our source data coordinator will contact you to discuss which figure panels we would need source data for and will also provide you with helpful tips on how to upload and organize the files.

The system will prompt you to fill in your funding and payment information. This will allow Wiley to send you a quote for the article processing charge (APC) in case of acceptance. This quote takes into account any reduction or fee waivers that you may be eligible for. Authors do not need to pay any fees before their manuscript is accepted and transferred to the publisher.

EMBO Press participates in many Publish and Read agreements that allow authors to publish Open Access with reduced/no publication charges. Check your eligibility: https://authorservices.wiley.com/author-resources/Journal-Authors/open-access/affiliation-policies-payments/index.html

As a matter of course, please make sure that you have correctly followed the instructions for authors as given on the submission website.

*** PLEASE NOTE *** As part of the EMBO Press transparent editorial process initiative (see our Editorial at https://dx.doi.org/10.1038/msb.2010.72), Molecular Systems Biology publishes online a Review Process File with each accepted manuscripts. This file will be published in conjunction with your paper and will include the anonymous referee reports, your point-by-point response and all pertinent correspondence relating to the manuscript. If you do NOT want this File to be

published, please inform the editorial office at msb@embo.org within 14 days upon receipt of the present letter.

Reviewer #1:

The manuscript by Kim and colleagues investigates how single-cell responses of bacteria to antibiotics (beta lactams) is connected to the population level response. This is an interesting question, as the bulk level time-kill curves are notoriously dynamic and non-monotonic over time and single cells are known to exhibit filamentation and then lysis-but the quantitative mapping from the latter to the former is unclear. The authors show that the probability of cell lysis increases with the extent of filamentation-and the microscopic parameters governing this relationship are strain- and dose-specific. In all cases, however, this filamentation-dependent lysis rate gives rise to population-level time-kill curves consistent with experimental observables (e.g. delayed killing and correlations between growth rate and kill rate).

Overall, I am quite positive about this work, which is technically sound and addresses a timely problem that will interest a broad range of readers in microbiology, biophysics, and quantitative biology. Among the paper's strengths are the powerful combination of mathematical modeling, single cell experiments, and classical time-kill experiments. The model is sufficiently simple that it can be described with a relatively small number of experimentally accessible effective parameters, and importantly, it can reproduce population-level observables (e.g. the time-delayed killing of beta lactams, the correlation between growth rate and lysis rate, and the bimodel length distribution of survivors) without additional free parameters.

I have mostly minor comments for the authors to consider.

The authors plot histograms of final cell length which, as the authors note, decays at large L because the longer cells have preferentially burst. From these histograms, they calculate P_L, which they refer to as the cumulative lysis probability. Are there potential artifacts from the fact that the long-L tail excludes cells that have burst? (In my understanding, converting these two quantities to a hazard function already accounts for this issue by estimating the real quantity of interest-the conditional probability of lysis at length L-but a bit of clarification might be helpful). Is it possible, or even potentially advantageous, to directly estimate this conditional probability from the single cell data? For example, could one bin the data to cells of a given length (interval) and directly assess the frequency of lysis in some interval? Perhaps a bit more discussion of the hazard function and the related theory of survival analysis would be helpful, but I leave it to the authors' discretion.

I think readers from outside of microbiology would benefit from a bit more general discussion of filamentation, perhaps in the intro or discussion. What (if anything) is known about why filamentation occurs, in general? Is it specific to rod-shaped and/or Gram negative bacteria? Are there (perhaps speculative) evolutionary reasons why filamentation might be advantageous, and/or does the current work offer insight into those questions? (The authors already touch on some of these points briefly in the discussion. See also my next two comments.)

I would recommend mentioning the bacterial species earlier, perhaps even in the abstract. Then one can speculate on generality later.

Related to the previous two points, I found myself wondering whether similar conceptual mechanisms might govern lysis even in cells that do not exhibit filamentation, but instead lyse when damage to the cell eclipses some "density of damage" threshold. I suppose I'm ultimately wondering whether filamentation (and therefore cell length), itself, is fundamental to the process or if, instead, the idea of damage-dependent lysis threshold would apply more generally. In that case, cell length (for this scenario) is essentially a surrogate for the density of damage.

I would recommend adding a section in the discussion about technical and conceptual limitations of the work. For example, on the conceptual side, the model is an obvious over-simplification of the underlying biophysics-and this is, in my view, largely a strength of the approach-but a brief discussion of limitations might add important context. It would also allow the authors to comment on the extent to which there are intuitive 'microscopic' explanations for some assumed features of the model (e.g. the fact that final length is described by a gamma distribution, as the authors mention, may relate to a physical model of cell wall cracking) but perhaps not for others. I'll note that, in any event, the absence of a microscopic model is not a big drawback, as the strength of the findings lies in the fact that measurements of single cell dynamics (whether parameterized by an abstract model or a mechanistic model) give rise to the population level responses.

While the trends in the data are often obvious, the authors should consider including formal statistical tests to strengthen the conclusions in any borderline cases (e.g. to demonstrate that dose-dependent changes in parameters are statistically significant).

I might consider adding the results Figure S5-at least in part-to the main text.

Please add number of cells to histogram plots (e.g. figure S6)

Reviewer #2:

This manuscript describes single-cell agarose-pad time-lapse measurements of bacterial filamentation in the presence of beta-lactam antibiotics (primarily carbenicillin). Measurements of the time of lysis reveal that lysis becomes more likely as filaments get longer, and this data is consistent with a simple model in which "damage" accumulates at a constant rate per unit of cell length, with a threshold amount of damage resulting in lysis. This model leads naturally to a correlation between growth rate and lysis rate (since fast-growing cells become longer faster and hence accumulate damage faster). A theoretical formulation is then presented which allows the single-cell observations to be used to predict population-level measurements (in the form of optical density vs time).

The topics addressed in this work - the interplay between antibiotic action and bacterial physiology, and the role of single-cell dynamics and cell morphology, and the connection to population-level dynamics and ultimately to clinical practice - are topical and interesting. However I have a number of concerns about both the manuscript and the study itself.

- The key observation, that lysis rate increases with filament length, is a very simple one, and it has a simple consequence that lysis rate correlates with growth rate. This is an interesting and insightful finding. Yet several of the other aspects of the study that are presented as important results are quite trivial. The parameters alpha, beta and H are presenting as having special significance, but they are just model fits and are bound to have different values for different strains and conditions. Also the integration of the single cell lysis curves into a population-level model, while nice, is not really of great significance, since one would expect any sensible model for single-cell lysis to be extendable into predictions at the population level. Finally, the statements about survivor length distributions being biphasic at the end of the manuscript are not really surprizing - the biphasic distribution simply arises because cells do not filament in the absence of antibiotic.

- The statements made in the introduction about the main results and significance of the study are vague: e.g. "These observations underscore the importance in quantifying key parameters of cellular lysis to couple with biomass dynamics of the population" and "Our results provide new insights into the mechanism of beta-lactam-induced cell lysis and a better understanding of the coupling between single-cell- and population-level dynamics". More specific statements about what new insights the study provides would sharpen the focus of the manuscript.

- The wording of the study implies that the findings apply to all beta-lactam antibiotics - although the vast majority of the experiments are done with carbenicillin, it is only rarely referred to. However, different beta-lactams target different protein components of the cell wall synthesis machinery, with different outcomes. Some beta-lactams do not cause filamentation at all. The authors should be careful about claiming that their findings are general to all beta-lactams.

- P.4, bottom of the page "for example..." this sentence is unclear. Does it just refer to one selected cell?

- Central to the analysis is the "lysis probability density" rhoL. This is the fraction of cells that lyse at a given final length - which of course depends on the length distribution of cells in the population. I find it very confusing to call it a probability density, since it is different from the intrinsic probability for a given cell to lyse.

- The key outcome of p4-5 is that longer filaments are more likely to lyse. But the distributions that are plotted in Figs 1 and 2 are hard to interpret because of the role played by the length distribution of cells in the population. The authors should compare these distributions to those of a null model in which lysis is not length-dependent. Moreover, error bars are missing from these distributions, which is important especially at long filament lengths where statistics are poor.

- Measurements of filament length (eg in Figure 2) are made only at the start and end points. This makes the plots of cell length rather misleading, since they look as though length is growing exponentially, but there is actually no intermediate data. It would have been helpful also to show how length changes as a function of time during filament growth.

- Caption of Figure 3c "Experimentally observed τE points qualitatively verify the boundary condition of τE." I do not understand what this means. What is the boundary condition of tauE? "Boundary condition" seems a confusing term to use, since it is usually found in a very different context.

- Two different theoretical formulations are presented for the shape of the lysis-length curves: the Hill formulation and the "mechanistic" formulation (presented in the supplement). It would make sense to fit the data with the Gamma distribution that emerges from the mechanistic model, and to show whether it is a better fit than the Hill function. But this is not done.

- Related to this, on p.5 it is stated that the Hill parameter H does not depend on antibiotic dose or temperature, but the numerical analysis suggests that H should increase with the square root of alpha. What does alpha correspond to

mechanistically, and is the prediction that H increases with it borne out by the experimental data?

- On p.5 equivalent observations are made about clinical strains, resulting in different parameter values. While it is nice that the study is extended beyond lab strains, I did not see the significance of this finding. Perhaps it would have been surprising if the parameter values had not been strain dependent?

- To my mind the most interesting part of the study is the mechanistic model, but this is not described at all in the main text. I find this an important deficiency, because the model is actually very simple, and without reading the supplement the reader cannot grasp the key insight of the paper - that a constant rate of damage per unit length can explain the length dependence of the lysis rate. Essentially the model just predicts a Poisson distribution for the amount of damage in a cell, and it would be helpful to have mentioned this.

- On p.6 it is stated "This model draws inspiration from cooperative binding in gene transcription activation, along with a physical model of cell wall crack formation from the accumulation of peptide defects". This sentence seems to me highly misleading. The model as presented in the supplement has nothing to do with transcription or crack formation. It is simply a Poisson model for damage accumulation and says nothing about the mechanism for the damage.

- On p.7 a stochastic model is introduced. Once again very little is said in the main text about this model, making it hard for the reader to assess the results. Actually this model is quite simple, but the authors should describe in simple terms in the main text how it works.

- On p.8 the connection between single cell lysis probability and population level dynamics is made via the mean filament length, i.e. the population heterogeneity in filament length plays no role. The authors should discuss under what conditions this assumption is valid - e.g. would it break down if the damage accumulation was non-linear in the filament length?

- The paragraph at the bottom of p.8-start of p.9 is hard to follow since the final two sentences do not follow either from each other or from what comes before in the paragraph.
•

- Throughout the manuscript, experimental data from OD measurements is used to make inferences about cell death (especially on p.9). Yet there are no actual measurements of live cells, which would require plating experiments. I find this an important omission - one cannot be sure that optical density is proportional to live biomass, as assumed here.

- One clear prediction of this model is that even at very low doses of antibiotic, there will eventually be lysis, since damage will accumulate to the threshold in the end. This could presumably be tested experimentally?

- On p.11 the authors talk extensively about biphasic survivor length distributions - "As such, for increasing antibiotic doses, we predicted that the average length of survivors would first increase and then decrease, that is, survivor length is biphasic". But this is really a trivial statement. In the absence of antibiotic, cells are short because they do not filament, while at high antibiotic they are short because the filaments have lysed. The fact that there is a peak at intermediate length is not really a new insight, it is to be expected if the lysis rate is length-dependent.

- On p.12-13 filamentation is presented as a potential beneficial mechanism by which the bacteria can survive antibiotic treatment. I find this argument tenuous. Bacteria filament because the antibiotic has disabled the division machinery, not because there is an "alternative option" that has been selected against by evolution.

- On p. 13 it is stated that "Since antibiotic resistance is often associated with the phenotypic heterogeneity-driven variance of single-cell lysis dynamics (Andrews, 2001; Artemova et al, 2015), H can be a useful metric in projecting the resistance emergence in a population". I also do not agree with this argument, since for beta-lactams, resistance mainly occurs through the production of beta-lactamase enzymes, rather than through modification of the physiology of the bacteria themselves.

Reviewer #3:

Review Kim et al. Mapping single-cell responses to population-level dynamics during antibiotic treatment

In their study, Kim et al. show using time-lapse microscopy that the lysis probability of E. coli during beta-lactam treatment depends on their filamentation length. They show that the cumulative lysis probability distribution can be described by a sigmoidal phenomenological curve. They further show that the critical length for lysis in this distribution depends on the antibiotic dose. In their interpretation, the cell wall accumulates a damage from antibiotics until the total reaches a threshold that triggers lysis. They then go on to predict temporal dynamics of population levels responses from this single cell response. A transient increase in total population biomass before an eventual decline can be explained by the continued elongation of cells before lysis. They argue that their model can explain the linear correlation between population-level growth rate and lysis rate. Finally,

they show that single-cell lysis kinetics successfully predicts the dose-response length of survivor cells for sublethal doses.

Overall, we really like this manuscript and think it is a solid price of work with many interesting and valuable observations that will be of interest to the microbial community. Although the model is phenomenological and does not address underlying molecular mechanisms, it makes successfully makes several impressive predictions. We think this work is elegant interesting and suitable for publication in MSB.

One minor concern is the linear correlation between maximum growth rate and lysis rate. It is not clear to what extent this prediction is backed up by experimental data. Moreover, the derivation is not clear in the main text and the reason for this correlation is not intuitive. Unless these points can be improved, to avoid confusing reader by mixing experimentally validated observations with pure simulation results, we suggest moving this figure to the Supplementary Information.

More specifically, Figure 4, is a theoretical prediction to draw the correlation between maximum growth and lysis rate at population level, but not backed up by experimental data. Authors should perform an experiment to show the predicted linearity between maximum growth rate (G) and lysis rate(D). This can be achieved by using minimal media with different carbon sources. As predicted in figure 4b, authors should select minimal media with three different carbon sources separately and determine the elongation rate in respective media. Also, a plot showing the experimental result as predicted in 4b, should be included.

Minor point:
In figure 5, panel b and c, no statistical significance is shown been 0 ug/ml Carb and different doses of Carb. Statistical significance of the means should be added.
For simulated cell length, no unit mentioned on Y axis, this should be added.

Point-by-point responses to reviewer comments

Reviewer #1:

The manuscript by Kim and colleagues investigates how single-cell responses of bacteria to antibiotics (beta lactams) is connected to the population level response. This is an interesting question, as the bulk level time-kill curves are notoriously dynamic and non-monotonic over time and single cells are known to exhibit filamentation and then lysis-but the quantitative mapping from the latter to the former is unclear. The authors show that the probability of cell lysis increases with the extent of filamentation-and the microscopic parameters governing this relationship are strain- and dose-specific. In all cases, however, this filamentation-dependent lysis rate gives rise to population-level time-kill curves consistent with experimental observables (e.g. delayed killing and correlations between growth rate and kill rate).

Overall, I am quite positive about this work, which is technically sound and addresses a timely problem that will interest a broad range of readers in microbiology, biophysics, and quantitative biology. Among the paper's strengths are the powerful combination of mathematical modeling, single cell experiments, and classical time-kill experiments. The model is sufficiently simple that it can be described with a relatively small number of experimentally accessible effective parameters, and importantly, it can reproduce population-level observables (e.g. the time-delayed killing of beta lactams, the correlation between growth rate and lysis rate, and the bimodal length distribution of survivors) without additional free parameters.

We thank the reviewer for the accurate and succinct summary of our work, as well as the appreciation of our approach. Below, we clarify the issues raised by the reviewer and revised the manuscript accordingly. We numbered the following questions for point-by-point responses.

I have mostly minor comments for the authors to consider.

Q1: The authors plot histograms of final cell length which, as the authors note, decays at large L because the longer cells have preferentially burst. From these histograms, they calculate P_L, which they refer to as the cumulative lysis probability. Are there potential artifacts from the fact that the long-L tail excludes cells that have burst? (In my understanding, converting these two quantities to a hazard function already accounts for this issue by estimating the real quantity of interest-the conditional probability of lysis at length L-but a bit of clarification might be helpful). Is it possible, or even potentially advantageous, to directly estimate this conditional probability from the single cell data? For example, could one bin the data to cells of a given length (interval) and directly assess the frequency of lysis in some interval? Perhaps a bit more discussion of the hazard function and the related theory of survival analysis would be helpful, but I leave it to the authors' discretion.

We thank the reviewer for pointing that hazard function, $P_H(L) = \frac{\rho_L}{1-P_L}$ (Eq. 2), accounts the conditional probability of lysis. As indicated by this equation, the hazard function formula is uniquely determined by the probability density, ρ_L. We determined ρ_L by binning the single-cell data, as suggested by the reviewer.

As long as ρ_L is determined accurately, there is no additional artifact in determining $P_H(L)$. Indeed, having a small ρ_L at long-L implies the rarity of such long cells due to lysis before reaching a long- L. In light of the reviewer's comment, we have elaborated on our description on how the hazard function is derived in the **Appendix Supplementary Methods**.

Q2: I think readers from outside of microbiology would benefit from a bit more general discussion of filamentation, perhaps in the intro or discussion. What (if anything) is known about why filamentation occurs, in general? Is it specific to rod-shaped and/or Gram negative bacteria? Are there (perhaps speculative)

evolutionary reasons why filamentation might be advantageous, and/or does the current work offer insight into those questions? (The authors already touch on some of these points briefly in the discussion. See also my next two comments.)

Thank you for the comment. A known mechanism of beta-lactam-induced filamentation is cell wall synthesis inhibition, when a beta-lactam binds to penicillin-binding proteins (PBPs) and inhibits peptidoglycan cross-linking (Cho *et al*, 2016; Chung *et al*, 2009; Vigouroux *et al*, 2020), while the cells continue to accumulate biomass (Choi *et al*, 2014; Rolinson, 1980a). As a result, individual cells elongate exponentially without division and become filamented until the accumulation of cross-link defects creates a pore where a bulge forms (Cushnie *et al*, 2016).

Filamentation has been reported in many rod-shaped Gram-negative and Gram-positive bacteria (Buijs *et al*, 2008; Cayron *et al*, 2020; Chen *et al*, 2005; Cushnie *et al.*, 2016; Elliott & Greenwood, 1983; Eng *et al*, 1985; Kjeldsen *et al*, 2015; Mason *et al*, 1995; Paulander *et al*, 2014).

It has been speculated that filamentation during beta-lactam antibiotic treatment can facilitate population recovery, upon antibiotic removal. In particular, a filamented cell can divide into multiple cells (Cayron *et al.*, 2020). When the antibiotic is removed before lysis occurs, the resulting cell number is roughly proportional to the filament length (Wehrens *et al*, 2018). Our work shows the condition under which filamentation can facilitate population recovery. As long as the antibiotic is removed before a cell reaches the critical length (L_c), a cell that filaments will generate more biomass on average will contribute more to biomass than a cell that does not filament or lyse.

To clarify the points above, we have revised our main text.

Q3: I would recommend mentioning the bacterial species earlier, perhaps even in the abstract. Then one can speculate on generality later.

Thank you for the suggestion, which we have incorporated.

Q4: Related to the previous two points, I found myself wondering whether similar conceptual mechanisms might govern lysis even in cells that do not exhibit filamentation, but instead lyse when damage to the cell eclipses some "density of damage" threshold. I suppose I'm ultimately wondering whether filamentation (and therefore cell length), itself, is fundamental to the process or if, instead, the idea of damage-dependent lysis threshold would apply more generally. In that case, cell length (for this scenario) is essentially a surrogate for the density of damage.

When *E. coli* cells are exposed to imipenem, they swell, rather than filament; thus, the overall cell size increases before lysis without division (Choi *et al.*, 2014). This evidence may suggest that the concept of damage accumulation in response to beta-lactam can be applicable to cells that do not form filaments, where the cell size can potentially serve as a surrogate for the total damage. As such, filamentation per se might not be a fundamental intermediate step leading toward cell death.

Q5: I would recommend adding a section in the discussion about technical and conceptual limitations of the work. For example, on the conceptual side, the model is an obvious over-simplification of the underlying biophysics-and this is, in my view, largely a strength of the approach-but a brief discussion of limitations might add important context. It would also allow the authors to comment on the extent to which there are intuitive 'microscopic' explanations for some assumed features of the model (e.g. the fact that final length

is described by a gamma distribution, as the authors mention, may relate to a physical model of cell wall cracking) but perhaps not for others. I'll note that, in any event, the absence of a microscopic model is not a big drawback, as the strength of the findings lies in the fact that measurements of single cell dynamics (whether parameterized by an abstract model or a mechanistic model) give rise to the population level responses.

Thank you for the suggestion. We have now incorporated an additional paragraph to discuss the technical and conceptual limitations. In particular, the reviewers' earlier questions on the role and generality of cell filamentation represent one such aspect.

Q6: While the trends in the data are often obvious, the authors should consider including formal statistical tests to strengthen the conclusions in any borderline cases (e.g. to demonstrate that dose-dependent changes in parameters are statistically significant).

Thank you for pointing out the needs of statistical significance in those figures. We have revised our figures and figure legends accordingly. Independent t-tests between non-treated to treated group in **Figure 5** showed extreme significance, P < 0.0001.

We also added correlation coefficient (c = - 0.9103) of fitted L_c and carbenicillin concentration in the **Figure 2B inset.**

Q7: I might consider adding the results Figure S5-at least in part-to the main text.

Thanks for the suggestion, we revised and included the figure as **EV1** (Expanded View 1) that will be shown in the main text. We minded putting two different models in the main figures to avoid conceptual confusion. We hope the revised format underscores the simple connection between single-cell and population level dynamics, while **EV1** allows easier access to our mechanistic model.

Q8: Please add number of cells to histogram plots (e.g. figure S6)

Thank you for the suggestion, we added the number of cells to the histogram plots in **Fig 2A**, **Appendix Fig S4**, **Appendix Fig S6,** and **Appendix Fig S9**.

Reviewer #2:

This manuscript describes single-cell agarose-pad time-lapse measurements of bacterial filamentation in the presence of beta-lactam antibiotics (primarily carbenicillin). Measurements of the time of lysis reveal that lysis becomes more likely as filaments get longer, and this data is consistent with a simple model in which "damage" accumulates at a constant rate per unit of cell length, with a threshold amount of damage resulting in lysis. This model leads naturally to a correlation between growth rate and lysis rate (since fast-growing cells become longer faster and hence accumulate damage faster). A theoretical formulation is then presented which allows the single-cell observations to be used to predict population-level measurements (in the form of optical density vs time).

The topics addressed in this work - the interplay between antibiotic action and bacterial physiology, and the role of single-cell dynamics and cell morphology, and the connection to population-level dynamics and ultimately to clinical practice - are topical and interesting. However, I have a number of concerns about both the manuscript and the study itself.

We are glad that the reviewer recognized the importance of mapping single-cell and population-level dynamics during antibiotic treatment. We appreciate the reviewer's constructive comments and suggestions, which we fully address below and in the revised manuscript.

Q1: The key observation, that lysis rate increases with filament length, is a very simple one, and it has a simple consequence that lysis rate correlates with growth rate. This is an interesting and insightful finding. Yet several of the other aspects of the study that are presented as important results are quite trivial. The parameters alpha, beta and H are presenting as having special significance, but they are just model fits and are bound to have different values for different strains and conditions. Also the integration of the single cell lysis curves into a population-level model, while nice, is not really of great significance, since one would expect any sensible model for single-cell lysis to be extendable into predictions at the population level. Finally, the statements about survivor length distributions being biphasic at the end of the manuscript are not really surprising - the biphasic distribution simply arises because cells do not filament in the absence of antibiotic.

We thank the reviewer for finding our key observation interesting and insightful. We regret the confusion regarding the rationale of the model choice for data fitting and for interpretation.

Indeed, from the experimental data, we deduced two parameters (L_c and H) to quantify the single-cell lysis dynamics. These two parameters are constrained by our choice of the model (Hill equation). This choice does not carry significance in terms of the quality of data fitting, nor does it imply a mechanism. However, the choice of this model simplifies the mapping between single-cell and population dynamics. For instance, using this formulation, L_c (along with cell growth rate) sets the time delay in population decline; and H approximately represents the proportionality between growth and lysis rates at the population level.

In general, a different equation (associated with different parameters) can be used to fit the experimentally derived P_L, but may not lead to the simple mapping described above.

We note that α and β are not fitted parameters. Instead, we proposed the damage-accumulation model as an attempt to explain how the apparent Hill equation (log-logistic distribution) can emerge. The damage-accumulation model leads to Gamma distribution in the lysis probability. This formulation does not allow intuitive analysis, but can be approximated by a log-logistic distribution (Eqs. S8-S11). By doing so, we can derive an analytic mapping between (α, β) and (L_c, H), whereas the latter allows a simple mapping to the population-level lysis dynamics.

We agree with the reviewer that any sensible single-cell model can be mapped to prediction of population dynamics. This can be done entirely by simulations or by analytical derivations; we showed both in our analysis. We believe that showing this mapping explicitly provides quantitative evidence for the following statement:

> *The population dynamics of beta-lactam-mediated lysis dynamics can be explained by the filamentation and lysis dynamics of single cells, despite the fact that the antibiotic cause various perturbations to individual cells at the molecular level.*

Without concrete experimental measurements, we do not feel there is *a priori* reason to believe the statement has to be correct.

Likewise, if we accept the measurements of the single-cell lysis dynamics (as quantified by the Hill equation), the biphasic dependence is expected. This expectation is consistent with experimental measurements by us (**Figure 5B-C**) and previous studies (Chung *et al*, 2012; Fredborg *et al*, 2015; McLaughlin & Sue, 2018; Oh *et al*, 2020; ROLINSON, 1980b). This consistency provides further support to the statement above.

Q2: The statements made in the introduction about the main results and significance of the study are vague: e.g. "These observations underscore the importance in quantifying key parameters of cellular lysis to couple with biomass dynamics of the population" and "Our results provide new insights into the mechanism of beta-lactam-induced cell lysis and a better understanding of the coupling between single-cell- and population-level dynamics". More specific statements about what new insights the study provides would sharpen the focus of the manuscript.

We regret the vagueness of our statements, which appears to also relate to the comments raised above (Q1). We hope that our response above will help to clarify the potential significance of our work. We have revised our introduction/discussion to better clarify this point.

Q3: The wording of the study implies that the findings apply to all beta-lactam antibiotics - although the vast majority of the experiments are done with carbenicillin, it is only rarely referred to. However, different beta-lactams target different protein components of the cell wall synthesis machinery, with different outcomes. Some beta-lactams do not cause filamentation at all. The authors should be careful about claiming that their findings are general to all beta-lactams.

Thank you for the suggestion, we have revised a sentence in the main text regarding the antibiotic type generalization to the following: "... we observed the same dependence of P_L on cell length when using two other beta-lactam antibiotics that exhibit the filamentation …"

Q4: P.4, bottom of the page "for example..." this sentence is unclear. Does it just refer to one selected cell?

Yes, we referred to one of the cells that we randomly selected to show in **Figure 1B**. We have revised the text to avoid confusion.

Q5: Central to the analysis is the "lysis probability density" rhoL. This is the fraction of cells that lyse at a given final length - which of course depends on the length distribution of cells in the population. I find it very

confusing to call it a probability density, since it is different from the intrinsic probability for a given cell to lyse.

We completely agree with the reviewer on the interpretation of ρ_L, as well as its distinction with the intrinsic probability for a given cell to lyse (P_H), though the latter can be derived from ρ_L (Eq. 2). We note that the standard statistics terminology for ρ_L is "probability density", whereas P_L corresponds to the cumulative distribution function.

Q6: The key outcome of p4-5 is that longer filaments are more likely to lyse. But the distributions that are plotted in Figs 1 and 2 are hard to interpret because of the role played by the length distribution of cells in the population. The authors should compare these distributions to those of a null model in which lysis is not length-dependent. Moreover, error bars are missing from these distributions, which is important especially at long filament lengths where statistics are poor.

We note that the distributions are overall reproducible between replicate experiments. To illustrate the variability between replicates, we have provided 95% confidence intervals for the empirical cumulative distribution functions.

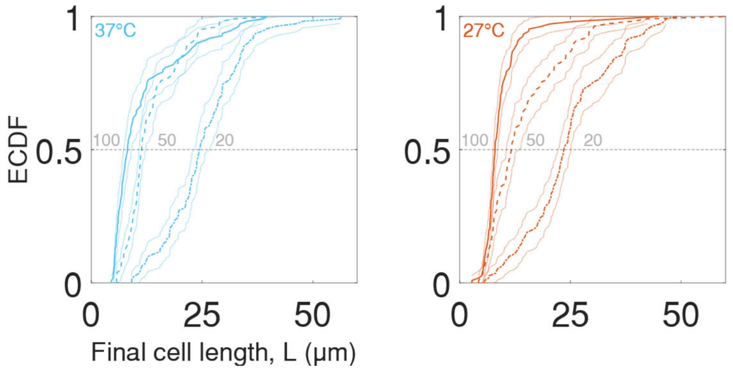

Figure R1. Confidence intervals of empirical cumulative distribution functions (ECDF). ECDF of the final length distributions were plotted in thicker lines, while 95% confidence intervals were plotted in thinner dotted lines using the MATLAB 'ecdf' function with 'Bound' option. Carbenicillin concentrations (μg/ml) were marked near the distributions.

Figure R1 has been incorporated to **Appendix Figure S3C** of the revised manuscript.

In addition, we analyzed cells in a different location (shown in green) in the same sample (**Figure 2A-B** Carbenicillin 20 μg/ml, shown in red below) to provide highly consistent fitted P_L achieved from the two imaging positions.

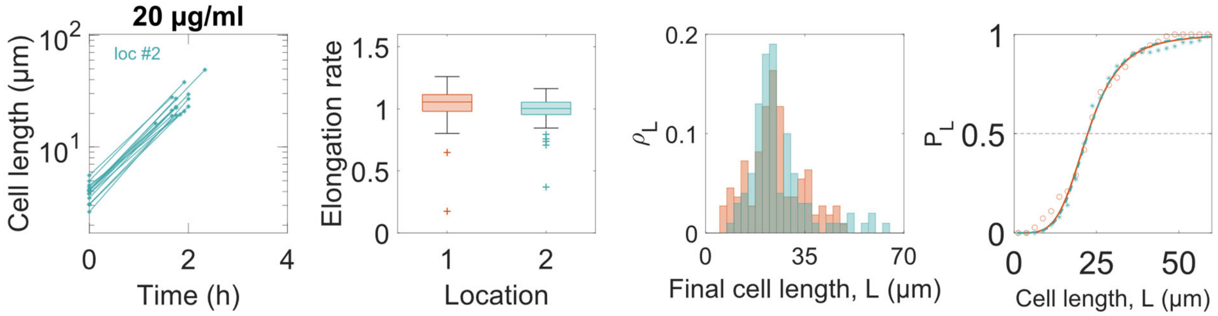

Figure R2. P_L was highly consistent in two imaging locations of one sample.

P_L of cells that were located in the second imaging locations of the same sample (Figure 2A, 20μg/ml, 37°C) showed highly consistent fitted P_L ($L_c = 22.55$, and $H = 4.71$).

Figure R2 has been incorporated to **Appendix Figure S3D** of the revised manuscript.

Theoretically, if lysis is completely independent of cell length, we would expect a *uniform distribution* for ρ_L and thus a linear P_L between a smallest possible cell and the largest possible cell. Our measurements show a drastic deviation from the uniform distribution and indicate cell length dependence.

Q7: Measurements of filament length (eg in Figure 2) are made only at the start and end points. This makes the plots of cell length rather misleading, since they look as though length is growing exponentially, but there is actually no intermediate data. It would have been helpful also to show how length changes as a function of time during filament growth.

Thank you for the suggestion. We note that single-cell exponential growth has been established (Wang *et al*, 2010). It is also established that this exponential growth continues even when cells were treated by beta-lactam antibiotics (Lee *et al*, 2016; Rolinson, 1980a; Yao *et al*, 2012).

In light of the reviewer's comment, however, we re-analyzed our raw data and tracked intermediate lengths of 16 cells (from **Figure 2**). One of these is that shown in **Figure 1B**, which we have updated as below. We also added a figure showing intermediate lengths of 15 other cells in **Figure R3** as **Appendix Figure S1**. We revised figure legends and the main text.

B

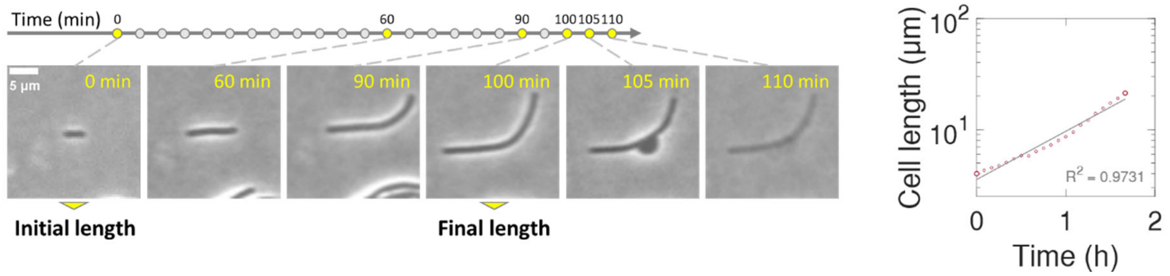

Figure 1B. Single-cell lengths were measured from time-lapse microscopy images upon beta-lactam antibiotic treatment.
Initial cell length was measured in the first time frame. Elongation ends with cell length shrinkage due to bulging. The longest length of each cell across the time frames was used as the final length. Tracked intermediate cell lengths were shown over time, while initial and final lengths were shown in larger circles. Linear regression of log-scaled lengths over time showed a constant rate of exponential elongation (solid gray line).

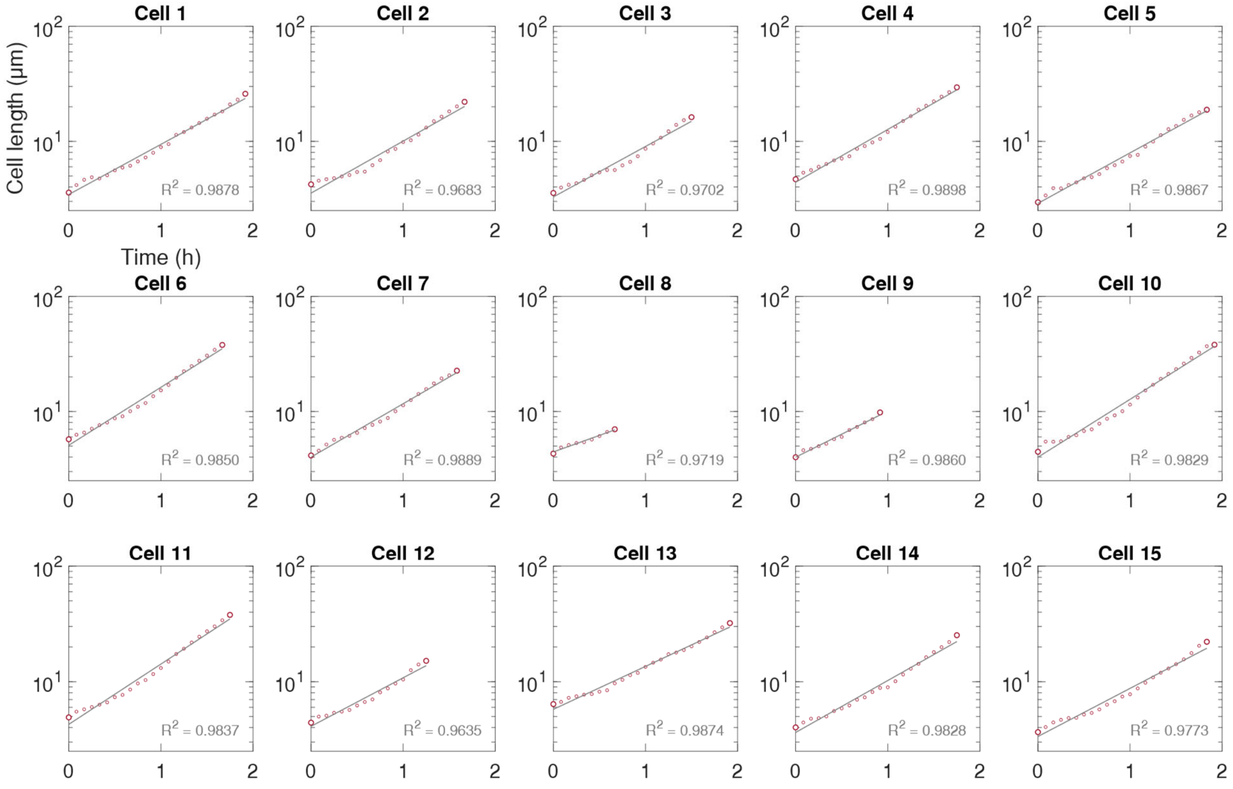

Figure R3. Intermediate cell lengths of beta-lactam antibiotic-induced filamentation.
The long-axis lengths of 15 additional cells were tracked as the one in **Figure 1B**. The initial and final lengths were marked in larger circles than the markers of intermediate lengths. Linear regression results on log-scaled cell length over time were shown in a solid gray line, supporting that cells elongate exponentially until burst.

Q8: Caption of Figure 3c "Experimentally observed $\boldsymbol{\tau E}$ points qualitatively verify the boundary condition of $\boldsymbol{\tau E}$." I do not understand what this means. What is the boundary condition of tauE? "Boundary condition" seems a confusing term to use, since it is usually found in a very different context.

We regret the confusion. We have revised the text to be clearer: “Experimental measurements confirm that τ_E decreases with the increase of antibiotic dose and growth rates.”

Q9: Two different theoretical formulations are presented for the shape of the lysis-length curves: the Hill formulation and the "mechanistic" formulation (presented in the supplement). It would make sense to fit the data with the Gamma distribution that emerges from the mechanistic model, and to show whether it is a better fit than the Hill function. But this is not done.

As noted in the response to Q1, the Hill formulation was empirical. However, it has the advantage of deriving a simple mapping between single-cell and population dynamics (Eq. 5). That is, our choice of the Hill formulation is based on two considerations:

(1) It provides an excellent fit with the experimental data (regardless of the underlying mechanisms).
(2) It enables an intuitive interpretation of some population-level parameters associated with beta-lactam responses (time delay, and the slope in the linear correlation between growth and lysis rates).

We agree with the reviewer that we can directly fit the Gamma distribution to the experimental data. Due to the complexity the mathematical formulation of Gamma distribution (relatively to Hill equation), that mapping will be more complex and less intuitive.

That being said, we showed that Gamma distribution can be approximately mapped to the Hill equation (see **Figure EV1**). Using analytic methods, we also showed that, the parameters of both distributions are approximately related by two equations: $L_c \approx \frac{\alpha}{\beta}$ and $H \approx \pi\sqrt{\frac{\alpha}{3}}$, (see **Appendix Supplementary Methods**). These two equations serve as a bridge to map the Gamma distributions to the population-level dynamics.

Q10: Related to this, on p.5 it is stated that the Hill parameter H does not depend on antibiotic dose or temperature, but the numerical analysis suggests that H should increase with the square root of alpha. What does alpha correspond to mechanistically, and is the prediction that H increases with it borne out by the experimental data?

In our damage accumulation model, α corresponds to the total number of damages that triggers burst and lysis. Indeed, H does not appear to strongly depend on antibiotic dose. By extension, according to our derivation ($H \approx \pi\sqrt{\frac{\alpha}{3}}$), we would expect not to strongly depend on antibiotic dose. If so, it implies that the extent by which a cell remains intact is dictated by the total damage it will experience. This notion makes intuitive sense, but future study is needed to provide experimental test.

Q11: On p.5 equivalent observations are made about clinical strains, resulting in different parameter values. While it is nice that the study is extended beyond lab strains, I did not see the significance of this finding. Perhaps it would have been surprising if the parameter values had not been strain dependent?

Before conducting the measurements, we do not have an *a priori* basis to expect one way or another. Indeed, if all different strains exhibit the same set of parameters, that would be surprising and interesting. That was not the case. A common theme emerging between different measurements is that **the same set of equations** (though not the same parameter values) can be used to describe the single-cell filamentation and lysis dynamics.

Q12: To my mind the most interesting part of the study is the mechanistic model, but this is not described at all in the main text. I find this an important deficiency, because the model is actually very simple, and without reading the supplement the reader cannot grasp the key insight of the paper - that a constant rate of damage per unit length can explain the length dependence of the lysis rate. Essentially the model just predicts a Poisson distribution for the amount of damage in a cell, and it would be helpful to have mentioned this.

We thank the reviewer for recognizing the value of the mechanistic model. We have now included the description of the rationale and formulation of the model in the main text, as part of **EV1** (Expanded View

1). We have revised the section in the *Mathematical model of damage accumulation* to incorporate the reviewer's suggestion (on describing the predicted Poisson distribution of damage accumulation).

Q13: On p.6 it is stated "This model draws inspiration from cooperative binding in gene transcription activation, along with a physical model of cell wall crack formation from the accumulation of peptide defects". This sentence seems to me highly misleading. The model as presented in the supplement has nothing to do with transcription or crack formation. It is simply a Poisson model for damage accumulation and says nothing about the mechanism for the damage.

We regret the confusion.

The reviewer is correct that our model does not deal with transcription. Our intended parallel was as follows. Cooperative activation of transcription by a TF can require the binding of the TF to multiple binding sites before triggering activation. In our model, cell lysis requires the accumulation of a certain amount of damage.

The reviewer is also correct that our damage accumulation model does not cover the *mechanism for the damage*. Instead, our model incorporates the notion that accumulation of cell-wall defects would lead to cell-wall crack formation before bursting (Huang *et al*, 2008; Yao *et al.*, 2012).

However, in the context of our paper, we recognized that this writing can be misleading. As such, we have removed the TF analogy and revised the paragraph to clarify.

Q14: On p.7 a stochastic model is introduced. Once again very little is said in the main text about this model, making it hard for the reader to assess the results. Actually, this model is quite simple, but the authors should describe in simple terms in the main text how it works.

We appreciate the suggestion. We added the following sentence to the section where we introduced stochastic model simulations:

"For each cell, we generated a random number between 0 to 1. The cell is set to be lysed (with no biomass contribution to the population) when its instantaneous lysis rate becomes larger than the number."

Q15: On p.8 the connection between single cell lysis probability and population level dynamics is made via the mean filament length, i.e. the population heterogeneity in filament length plays no role. The authors should discuss under what conditions this assumption is valid - e.g. would it break down if the damage accumulation was non-linear in the filament length?

We did not intend to imply the exclusion of the role of heterogeneity in our deterministic model. It is an approximation that allows the derivation of the analytical solution on time delay. Despite this simplification, our results show that the deterministic model matches the stochastic model well (**Appendix Figure S7**).

This deterministic model does not rely on the damage-accumulation model. As such, how the damage assumption depends on the cell length is not related to the effectiveness of the mean-field approximation.

Q16: The paragraph at the bottom of p.8-start of p.9 is hard to follow since the final two sentences do not follow either from each other or from what comes before in the paragraph.

Thanks for the comment. We have revised our main text to improve clarity and rigor:

“This deterministic model allows us to compute how the total biomass changes as a function of time with the average parameters of single cells (**Figure 3A** and **Appendix Figure S7**).”

Q17: Throughout the manuscript, experimental data from OD measurements is used to make inferences about cell death (especially on p.9). Yet there are no actual measurements of live cells, which would require plating experiments. I find this an important omission - one cannot be sure that optical density is proportional to live biomass, as assumed here.

Thank you for the comment. The population measurements using OD were intended to track the total biomass of the culture, not the number of live cells. Both the time-delay in the onset of population decline and the correlation between growth and lysis rates have been established using biomass measurements.

Plating does not allow reliable measurement of the living biomass: a highly filamented cell has much more biomass than a short cell, but each will lead to a single colony. On the technical side, plating is low throughput and error-prone; it is impractical to obtain the temporal population dynamics with high temporal resolution and high accuracy.

Q18: One clear prediction of this model is that even at very low doses of antibiotic, there will eventually be lysis since damage will accumulate to the threshold in the end. This could presumably be tested experimentally?

We regret the confusion. Our model does not predict cells would lyse even at very low antibiotic doses. Instead, at a very low dose of the antibiotic (e.g. much below the lethal dose), cells may not suffer from any damage at all (i.e., β=0).

Q19: On p.11 the authors talk extensively about biphasic survivor length distributions - "As such, for increasing antibiotic doses, we predicted that the average length of survivors would first increase and then decrease, that is, survivor length is biphasic". But this is really a trivial statement. In the absence of antibiotic, cells are short because they do not filament, while at high antibiotic they are short because the filaments have lysed. The fact that there is a peak at intermediate length is not really a new insight, it is to be expected if the lysis rate is length-dependent.

We regret the confusion. As we mentioned in the last paragraph of the first question, we are not claiming the biphasic trend itself to be novel. As noted by the reviewer, it is an expected consequence of the length-dependent lysis probability. The consistency between the prediction and experimental observation provides additional support for the following statement:

> *The population dynamics of beta-lactam-mediated lysis dynamics can be explained by the filamentation and lysis dynamics of single cells, despite the fact that the antibiotic causes various perturbations to individual cells at the molecular level.*

Q20: On p.12-13 filamentation is presented as a potential beneficial mechanism by which the bacteria can survive antibiotic treatment. I find this argument tenuous. Bacteria filament because the antibiotic has disabled the division machinery, not because there is an "alternative option" that has been selected against by evolution.

Thank you for the comment. To clarify, the contrast is between two scenarios when cells are treated with a beta-lactam antibiotic:

1) Cells continue to grow (without division), with a risk of bursting.
2) Cells stop growth or division, with a drastically reduced risk of death.

The second scenario corresponds to the trait of persisters. It has been demonstrated that a filamented cell can divide into multiple daughter cells after antibiotic removal (Cayron *et al.*, 2020; Wehrens *et al.*, 2018). If the antibiotic is removed before a cell reaches L_c, cells that filament may contribute more to biomass recovery than cells that do not grow. If so, filamentation can be a potentially beneficial choice, relative to no growth. Indeed, being exposed to an antibiotic is detrimental to a bacterial population. Being able to filament can potentially alleviate this effect.

In light of the reviewer's comment, we have made our writing more specific to avoid confusion or any impression of over-claiming.

Q21: On p. 13 it is stated that "Since antibiotic resistance is often associated with the phenotypic heterogeneity-driven variance of single-cell lysis dynamics (Andrews, 2001; Artemova et al, 2015), H can be a useful metric in projecting the resistance emergence in a population". I also do not agree with this argument, since for beta-lactams, resistance mainly occurs through the production of beta-lactamase enzymes, rather than through modification of the physiology of the bacteria themselves.

Thank you for the comment. We realize that this sentence was potentially misleading; we have thus removed it in the revised manuscript and revised the paragraph accordingly.

To clarify, H measures the steepness of the response curve in the cumulative lysis probability and reflects variations in lysis length and time between cells. If the H is small, the cell-to-cell phenotypic heterogeneity can be increased due to different lengths without lysis. Such heterogeneity has been shown to affect the emergence of new antibiotic resistance.

Reviewer #3:

In their study, Kim et al. show using time-lapse microscopy that the lysis probability of *E. coli* during beta-lactam treatment depends on their filamentation length. They show that the cumulative lysis probability distribution can be described by a sigmoidal phenomenological curve. They further show that the critical length for lysis in this distribution depends on the antibiotic dose. In their interpretation, the cell wall accumulates a damage from antibiotics until the total reaches a threshold that triggers lysis. They then go on to predict temporal dynamics of population levels responses from this single cell response. A transient increase in total population biomass before an eventual decline can be explained by the continued elongation of cells before lysis. They argue that their model can explain the linear correlation between population-level growth rate and lysis rate. Finally, they show that single-cell lysis kinetics successfully predicts the dose-response length of survivor cells for sublethal doses.

Overall, we really like this manuscript and think it is a solid price of work with many interesting and valuable observations that will be of interest to the microbial community. Although the model is phenomenological and does not address underlying molecular mechanisms, it makes successfully makes several impressive predictions. We think this work is elegant interesting and suitable for publication in MSB.

One minor concern is the linear correlation between maximum growth rate and lysis rate. It is not clear to what extent this prediction is backed up by experimental data. Moreover, the derivation is not clear in the main text and the reason for this correlation is not intuitive. Unless these points can be improved, to avoid confusing reader by mixing experimentally validated observations with pure simulation results, we suggest moving this figure to the Supplementary Information.

More specifically, Figure 4, is a theoretical prediction to draw the correlation between maximum growth and lysis rate at population level, but not backed up by experimental data. Authors should perform an experiment to show the predicted linearity between maximum growth rate (G) and lysis rate(D). This can be achieved by using minimal media with different carbon sources. As predicted in figure 4b, authors should select minimal media with three different carbon sources separately and determine the elongation rate in respective media. Also, a plot showing the experimental result as predicted in 4b, should be included.

We regret the confusion. The linear correlation between G and D at the population level has been previously demonstrated (Lee *et al*, 2018; Tuomanen *et al*, 1986). In these studies, the growth rates were modulated in several ways, including adjusting the flow rate in a chemostat (Tuomanen *et al.*, 1986) and changing media or temperature (Lee *et al.*, 2018). Our single-cell measurements provide an explanation for these observations (**Figure 4**).

We present Eq. 8 in **Methods** to show a detailed derivation of Eq. 5 in the main text. Though we have further illustrations of the proportionality between G and D from Eq. 5 on page 9, we noticed that the confusion might have arisen from not showing it as an equation. We added a short sentence, "That is, $D = HG$." to the main text to clarify the linearity.

Minor point:

In figure 5, panel b and c, no statistical significance is shown been 0 ug/ml Carb and different doses of Carb. Statistical significance of the means should be added. For simulated cell length, no unit mentioned on Y axis, this should be added.

We thank the reviewer for pointing out the need for statistical analysis in **Figure 5**. We added the statistical significance and revised the figure legend accordingly. We also added (a.u.) for the Y-axis units.

References

Buijs J, Dofferhoff ASM, Mouton JW, Wagenvoort JHT, van der Meer JWM (2008) Concentration-dependency of β-lactam-induced filament formation in Gram-negative bacteria. *Clinical Microbiology and Infection* 14: 344-349

Cayron J, Dedieu A, Lesterlin C (2020) Bacterial filament division dynamics allows rapid post-stress cell proliferation. *bioRxiv*: 2020.2003.2016.993345

Chen K, Sun GW, Chua KL, Gan YH (2005) Modified virulence of antibiotic-induced Burkholderia pseudomallei filaments. *Antimicrob Agents Chemother* 49: 1002-1009

Cho H, Wivagg CN, Kapoor M, Barry Z, Rohs PDA, Suh H, Marto JA, Garner EC, Bernhardt TG (2016) Bacterial cell wall biogenesis is mediated by SEDS and PBP polymerase families functioning semi-autonomously. *Nature Microbiology* 1: 16172

Choi J, Yoo J, Lee M, Kim E-G, Lee JS, Lee S, Joo S, Song SH, Kim E-C, Lee JC *et al* (2014) A rapid antimicrobial susceptibility test based on single-cell morphological analysis. *Science Translational Medicine* 6: 267ra174-267ra174

Chung C-C, Cheng IF, Chen H-M, Kan H-C, Yang W-H, Chang H-C (2012) Screening of Antibiotic Susceptibility to β-Lactam-Induced Elongation of Gram-Negative Bacteria Based on Dielectrophoresis. *Analytical Chemistry* 84: 3347-3354

Chung HS, Yao Z, Goehring NW, Kishony R, Beckwith J, Kahne D (2009) Rapid beta-lactam-induced lysis requires successful assembly of the cell division machinery. *Proceedings of the National Academy of Sciences of the United States of America* 106: 21872-21877

Cushnie TPT, O'Driscoll NH, Lamb AJ (2016) Morphological and ultrastructural changes in bacterial cells as an indicator of antibacterial mechanism of action. *Cellular and Molecular Life Sciences* 73: 4471-4492

Elliott TSJ, Greenwood D (1983) The response of Pseudomonas aeruginosa to azlocillin, ticarcillin and cefsulodin. *Journal of Medical Microbiology* 16: 351-362

Eng RH, Cherubin C, Smith SM, Buccini F (1985) Inoculum effect of beta-lactam antibiotics on Enterobacteriaceae. *Antimicrobial agents and chemotherapy* 28: 601-606

Fredborg M, Rosenvinge FS, Spillum E, Kroghsbo S, Wang M, Sondergaard TE (2015) Automated image analysis for quantification of filamentous bacteria. *BMC microbiology* 15: 255-255

Huang KC, Mukhopadhyay R, Wen B, Gitai Z, Wingreen NS (2008) Cell shape and cell-wall organization in Gram-negative bacteria. *Proceedings of the National Academy of Sciences* 105: 19282-19287

Kjeldsen TSB, Sommer MOA, Olsen JE (2015) Extended spectrum β-lactamase-producing Escherichia coli forms filaments as an initial response to cefotaxime treatment. *BMC microbiology* 15: 63-63

Lee AJ, Wang S, Meredith HR, Zhuang B, Dai Z, You L (2018) Robust, linear correlations between growth rates and beta-lactam-mediated lysis rates. *Proceedings of the National Academy of Sciences of the United States of America* 115: 4069-4074

Lee TK, Meng K, Shi H, Huang KC (2016) Single-molecule imaging reveals modulation of cell wall synthesis dynamics in live bacterial cells. *Nature Communications* 7: 13170

Mason DJ, Power EG, Talsania H, Phillips I, Gant VA (1995) Antibacterial action of ciprofloxacin. *Antimicrob Agents Chemother* 39: 2752-2758

McLaughlin HP, Sue D (2018) Rapid antimicrobial susceptibility testing and β-lactam-induced cell morphology changes of Gram-negative biological threat pathogens by optical screening. *BMC microbiology* 18: 218-218

Oh J, Ryu JS, Lee M, Jung J, Han S, Chung HJ, Park Y (2020) Three-dimensional label-free observation of individual bacteria upon antibiotic treatment using optical diffraction tomography. *Biomed Opt Express* 11: 1257-1267

Paulander W, Wang Y, Folkesson A, Charbon G, Løbner-Olesen A, Ingmer H (2014) Bactericidal antibiotics increase hydroxyphenyl fluorescein signal by altering cell morphology. *PloS one* 9: e92231-e92231

Rolinson GN (1980a) Effect of -Lactam Antibiotics on Bacterial Cell Growth Rate. *Microbiology* 120: 317-323

ROLINSON GN (1980b) Effect of β-Lactam Antibiotics on Bacterial Cell Growth Rate. *Microbiology* 120: 317-323

Tuomanen E, Cozens R, Tosch W, Zak O, Tomasz A (1986) The rate of killing of Escherichia coli by beta-lactam antibiotics is strictly proportional to the rate of bacterial growth. *Journal of general microbiology* 132: 1297-1304

Vigouroux A, Cordier B, Aristov A, Alvarez L, Özbaykal G, Chaze T, Oldewurtel ER, Matondo M, Cava F, Bikard D *et al* (2020) Class-A penicillin binding proteins do not contribute to cell shape but repair cell-wall defects. *eLife* 9: e51998

Wang P, Robert L, Pelletier J, Dang WL, Taddei F, Wright A, Jun S (2010) Robust Growth of Escherichia coli. *Current Biology* 20: 1099-1103

Wehrens M, Ershov D, Rozendaal R, Walker N, Schultz D, Kishony R, Levin PA, Tans SJ (2018) Size Laws and Division Ring Dynamics in Filamentous Escherichia coli cells. *Current biology : CB* 28: 972-979.e975

Yao Z, Kahne D, Kishony R (2012) Distinct single-cell morphological dynamics under beta-lactam antibiotics. *Mol Cell* 48: 705-712

24th Apr 2023

Manuscript Number: MSB-2022-11475R
Title: Mapping single-cell responses to population-level dynamics during antibiotic treatment

Dear Lingchong,

Thank you for sending us your revised manuscript. My colleague Jingyi Hou is on maternity leave and I took over handling your manuscript. We have now heard back from two of the three reviewers who were asked to evaluate your revised study. Unfortunately, reviewer #2 has not yet sent us their comments. In the interest of time, and as the recommendations of reviewer #1 and #3 are similar we decided to proceed with these two available reports. As you will see below, both reviewers are satisfied with the performed revisions and support publication.

Before we formally accept the study for publication, we would ask you to address some remaining editorial issues listed below.

- Our data editors have noted some missing information in the figure legends, please see the attached .doc file. Please make all requested text changes using the attached file and *keeping the "track changes" mode* so that we can easily access the edits made.

- Please provide 5 keywords.

- Please include callouts for: Figure EV1B, Appendix Figure S2A-C, Appendix Figure S3B-D, Appendix Figure S6A and S6C.

- The callout for Table S1 should be renamed to Appendix Table S1.

- In the Appendix file, the Figures and Tables should be labeled: Appendix Figure S1-S9 and Appendix Table S1.

Please resubmit your revised manuscript online **within one month** and ideally as soon as possible. If we do not receive the revised manuscript within this time period, the file might be closed and any subsequent resubmission would be treated as a new manuscript. Please use the Manuscript Number (above) in all correspondence.

Click on the link below to submit your revised paper.

https://msb.msubmit.net/cgi-bin/main.plex

As a matter of course, please make sure that you have correctly followed the instructions for authors as given on the submission website.

Thank you for submitting this paper to Molecular Systems Biology.

Kind regards,

Maria

Maria Polychronidou, PhD
Senior Editor
Molecular Systems Biology

If you do choose to resubmit, please click on the link below to submit the revision online before 24th May 2023.

https://msb.msubmit.net/cgi-bin/main.plex

IMPORTANT:
Please note that corresponding authors are required to supply an ORCID ID for their name upon submission of a revised manuscript (EMBO Press signed a joint statement to encourage ORCID adoption).
(https://www.embopress.org/page/journal/17444292/authorguide#editorialprocess)
Currently, our records indicate that the ORCID for your account is 0000-0003-3725-4007.

Please click the link below to modify this ORCID:

Link Not Available

The system will prompt you to fill in your funding and payment information. This will allow Wiley to send you a quote for the article processing charge (APC) in case of acceptance. This quote takes into account any reduction or fee waivers that you may be eligible for. Authors do not need to pay any fees before their manuscript is accepted and transferred to the publisher.

*** PLEASE NOTE *** As part of the EMBO Press transparent editorial process initiative (see our Editorial at https://dx.doi.org/10.1038/msb.2010.72 , Molecular Systems Biology will publish online a Review Process File to accompany accepted manuscripts. When preparing your letter of response, please be aware that in the event of acceptance, your cover letter/point-by-point document will be included as part of this File, which will be available to the scientific community. More information about this initiative is available in our Instructions to Authors. If you have any questions about this initiative, please contact the editorial office (msb@embo.org).

Reviewer #1:

The authors have improved an already strong manuscript with a number of additions and clarifications that improve readability, place the work in context, and appropriately address potential limitations raised by all reviewers.

Reviewer #3:

In this current version of manuscript, authors sufficiently addressed our concerns and revised manuscript is well written. Therefore, we recommend this work for publication in molecular systems biology

ll editorial and formatting issues were resol ed by the authors.

25th Apr 2023

Manuscript number: MSB-2022-11475RR
Title: Mapping single-cell responses to population-level dynamics during antibiotic treatment

Dear Lingchong,

Thank you again for sending us your revised manuscript. We are now satisfied with the modifications made and I am pleased to inform you that your paper has been accepted for publication.

*** PLEASE NOTE *** As part of the EMBO Publications transparent editorial process initiative (see our Editorial at https://dx.doi.org/10.103/msb.2010.72), Molecular Systems Biology publishes online a Review Process File with each accepted manuscripts. This file will be published in conjunction with your paper and will include the anonymous referee reports, your point- by-point response and all pertinent correspondence relating to the manuscript. If you do NOT want this File to be published, please inform the editorial office at msb@embo.org within 14 days upon receipt of the present letter.

LICENSE AND PAYMENT:
All articles published in Molecular Systems Biology are fully open access: immediately and freely available to read, download and share.

Molecular Systems Biology charges an article processing charge (APC) to cover the publication costs. You, as the corresponding author for this manuscript, should have already received a quote with the article processing fee separately. Please let us know in case this quote has not been received.

Once your article is at Wiley for editorial production, you will receive an email from Wiley's Author Services system, which will ask you to log in and will present you with the publication license form for completion. Within the same system the publication fee can be paid by credit card, an invoice or pro forma can be requested.

Payment of the publication charge and the signed Open Access Agreement form must be received before the article can be published online.

Upon acceptance it is mandatory for you to return the completed payment form. Failure to send back the form may result in a delay in the publication of your paper.

Molecular Systems Biology articles are published under the Creative Commons licence CC BY, which facilitates the sharing of scientific information by reducing legal barriers, while mandating attribution of the source in accordance to standard scholarly practice.

Proofs will be forwarded to you within the next 2-3 weeks.

Thank you very much for submitting your work to Molecular Systems Biology.

Kind regards,

Maria

Maria Polychronidou, PhD
Senior Editor
Molecular Systems Biology

EMBO Press Author Checklist

Corresponding Author Name: Lingchong You	
Journal Submitted to: Molecular Systems Biology	
Manuscript Number: MSB 2022-11475	

USEFUL LINKS FOR COMPLETING THIS FORM
The EMBO Journal - Author Guidelines
EMBO Reports - Author Guidelines
Molecular Systems Biology - Author Guidelines
EMBO Molecular Medicine - Author Guidelines

Reporting Checklist for Life Science Articles (updated January
This checklist is adapted from Materials Design Analysis Reporting (MDAR) Checklist for Authors. MDAR establishes a minimum set of requirements in transparent reporting in the life sciences (see Statement of Task: 10.31222/osf.io/9sm4x). Please follow the journal's guidelines in preparing your
Please note that a copy of this checklist will be published alongside your article.

Abridged guidelines for figures
1. Data
The data shown in figures should satisfy the following conditions:
- the data were obtained and processed according to the field's best practice and are presented to reflect the results of the experiments in an accurate and unbiased manner.
- ideally, figure panels should include only measurements that are directly comparable to each other and obtained with the same assay.
- plots include clearly labeled error bars for independent experiments and sample sizes. Unless justified, error bars should not be shown for technical
- if n<5, the individual data points from each experiment should be plotted. Any statistical test employed should be justified.
- Source Data should be included to report the data underlying figures according to the guidelines set out in the authorship guidelines on Data

2. Captions
Each figure caption should contain the following information, for each panel where they are relevant:
- a specification of the experimental system investigated (eg cell line, species name).
- the assay(s) and method(s) used to carry out the reported observations and measurements.
- an explicit mention of the biological and chemical entity(ies) that are being measured.
- an explicit mention of the biological and chemical entity(ies) that are altered/varied/perturbed in a controlled manner.
- the exact sample size (n) for each experimental group/condition, given as a number, not a range;
- a description of the sample collection allowing the reader to understand whether the samples represent technical or biological replicates (including how many animals, litters, cultures, etc.).
- a statement of how many times the experiment shown was independently replicated in the laboratory.
- definitions of statistical methods and measures:
  - common tests, such as t-test (please specify whether paired vs. unpaired), simple χ2 tests, Wilcoxon and Mann-Whitney tests, can be unambiguously identified by name only, but more complex techniques should be described in the methods section;
  - are tests one-sided or two-sided?
  - are there adjustments for multiple comparisons?
  - exact statistical test results, e.g., P values = x but not P values < x;
  - definition of 'center values' as median or average;
  - definition of error bars as s.d. or s.e.m.

Please complete ALL of the questions below.
Select "Not Applicable" only when the requested information is not relevant for your study.

Materials

Newly Created Materials	Information included in the manuscript?	In which section is the information available? (Reagents and Tools Table, Materials and Methods, Figures, Data Availability Section)
New materials and reagents need to be available; do any restrictions apply?	Not Applicable	

Antibodies	Information included in the manuscript?	In which section is the information available? (Reagents and Tools Table, Materials and Methods, Figures, Data Availability Section)
For **antibodies** provide the following information:<br>- Commercial antibodies: RRID (if possible) or supplier name, catalogue number and or/clone number<br>- Non-commercial: RRID or citation	Not Applicable	

DNA and RNA sequences	Information included in the manuscript?	In which section is the information available? (Reagents and Tools Table, Materials and Methods, Figures, Data Availability Section)
**Short novel DNA or RNA including primers, probes**: provide the sequences.	Not Applicable	

Cell materials	Information included in the manuscript?	In which section is the information available? (Reagents and Tools Table, Materials and Methods, Figures, Data Availability Section)
**Cell lines:** Provide species information, strain. Provide accession number in repository **OR** supplier name, catalog number, clone number, and/**OR** RRID.	Not Applicable	
**Primary cultures:** Provide species, strain, sex of origin, genetic modification status.	Not Applicable	
Report if the cell lines were recently **authenticated** (e.g., by STR profiling) and tested for mycoplasma contamination.	Not Applicable	

Experimental animals	Information included in the manuscript?	In which section is the information available? (Reagents and Tools Table, Materials and Methods, Figures, Data Availability Section)
**Laboratory animals or Model organisms:** Provide species, strain, sex, age, genetic modification status. Provide accession number in repository **OR** supplier name, catalog number, clone number, **OR** RRID.	Not Applicable	
**Animal observed in or captured from the field:** Provide species, sex, and age where possible.	Not Applicable	
Please detail **housing and husbandry conditions**.	Not Applicable	

Plants and microbes	Information included in the manuscript?	In which section is the information available? (Reagents and Tools Table, Materials and Methods, Figures, Data Availability Section)
**Plants:** provide species and strain, ecotype and cultivar where relevant, unique accession number if available, and source (including location for collected wild specimens).	Not Applicable	
**Microbes:** provide species and strain, unique accession number if available, and source.	Yes	Materials and Methods

Human research participants	Information included in the manuscript?	In which section is the information available? (Reagents and Tools Table, Materials and Methods, Figures, Data Availability Section)
If collected and within the bounds of privacy constraints report on age, sex and gender or ethnicity for all study participants.	Not Applicable	

Core facilities	Information included in the manuscript?	In which section is the information available? (Reagents and Tools Table, Materials and Methods, Figures, Data Availability Section)
If your work benefited from core facilities, was their service mentioned in the acknowledgments section?	Not Applicable	

Design

Study protocol	Information included in the manuscript?	In which section is the information available? (Reagents and Tools Table, Materials and Methods, Figures, Data Availability Section)
If study protocol has been **pre-registered, provide DOI in the manuscript**. For clinical trials, provide the trial registration number **OR** cite DOI.	Not Applicable	
Report the **clinical trial registration number** (at ClinicalTrials.gov or equivalent), where applicable.	Not Applicable	

Laboratory protocol	Information included in the manuscript?	In which section is the information available? (Reagents and Tools Table, Materials and Methods, Figures, Data Availability Section)
Provide DOI OR other citation details if **external detailed step-by-step protocols** are available.	Not Applicable	

Experimental study design and statistics	Information included in the manuscript?	In which section is the information available? (Reagents and Tools Table, Materials and Methods, Figures, Data Availability Section)
Include a statement about **sample size** estimate even if no statistical methods were used.	Yes	Figures
Were any steps taken to minimize the effects of subjective bias when allocating animals/samples to treatment (e.g. **randomization procedure**)? If yes, have they been described?	Not Applicable	
Include a statement about **blinding** even if no blinding was done.	Not Applicable	
Describe **inclusion/exclusion criteria** if samples or animals were excluded from the analysis. Were the criteria pre-established? If sample or data points were omitted from analysis, report if this was due to attrition or intentional exclusion and provide justification.	Not Applicable	
For every figure, are **statistical tests** justified as appropriate? Do the data meet the assumptions of the tests (e.g., normal distribution)? Describe any methods used to assess it. Is there an estimate of variation within each group of data? Is the variance similar between the groups that are being statistically compared?	Yes	Materials and Methods, Figures

Sample definition and in-laboratory replication	Information included in the manuscript?	In which section is the information available? (Reagents and Tools Table, Materials and Methods, Figures, Data Availability Section)
In the figure legends: state number of times the experiment was **replicated** in laboratory.	Yes	Figures
In the figure legends: define whether data describe **technical or biological replicates**.	Yes	Figures

Ethics

Ethics	Information included in the manuscript?	In which section is the information available? (Reagents and Tools Table, Materials and Methods, Figures, Data Availability Section)
Studies involving **human participants**: State details of **authority granting ethics approval** (IRB or equivalent committee(s), provide reference number for approval.	Not Applicable	
Studies involving **human participants**: Include a statement confirming that **informed consent** was obtained from all subjects and that the experiments conformed to the principles set out in the WMA Declaration of Helsinki and the Department of Health and Human Services Belmont Report.	Not Applicable	
Studies involving **human participants:** For publication of **patient photos**, include a statement confirming that consent to publish was obtained.	Not Applicable	
Studies involving experimental **animals**: State details of **authority granting ethics approval** (IRB or equivalent committee(s), provide reference number for approval. Include a statement of compliance with ethical regulations.	Not Applicable	
Studies involving **specimen and field samples:** State if relevant **permits** obtained, provide details of authority approving study; if none were required, explain why.	Not Applicable	

Dual Use Research of Concern (DURC)	Information included in the manuscript?	In which section is the information available? (Reagents and Tools Table, Materials and Methods, Figures, Data Availability Section)
Could your study fall under dual use research restrictions? Please check biosecurity documents and list of **select agents and toxins** (CDC): https://www.selectagents.gov/sat/list.htm	Not Applicable	
If you used a select agent, is the security level of the lab appropriate and reported in the manuscript?	Not Applicable	
If a study is subject to dual use research of concern regulations, is the name of the **authority granting approval and reference number** for the regulatory approval provided in the manuscript?	Not Applicable	

Reporting

The MDAR framework recommends adoption of discipline-specific guidelines, established and endorsed through community initiatives. Journals have their own policy about requiring specific guidelines and recommendations to complement MDAR.

Adherence to community standards	Information included in the manuscript?	In which section is the information available? (Reagents and Tools Table, Materials and Methods, Figures, Data Availability Section)
State if relevant guidelines or checklists (e.g., **ICMJE, MIBBI, ARRIVE, PRISMA**) have been followed or provided.	Not Applicable	
For **tumor marker prognostic studies,** we recommend that you follow the **REMARK** reporting guidelines (see link list at top right). See author guidelines, under 'Reporting Guidelines'. Please confirm you have followed these guidelines.	Not Applicable	
For **phase II and III randomized controlled trials**, please refer to the **CONSORT** flow diagram (see link list at top right) and submit the CONSORT checklist (see link list at top right) with your submission. See author guidelines, under 'Reporting Guidelines'. Please confirm you have submitted this list.	Not Applicable	

Data Availability

Data availability	Information included in the manuscript?	In which section is the information available? (Reagents and Tools Table, Materials and Methods, Figures, Data Availability Section)
Have **primary datasets** been deposited according to the journal's guidelines (see 'Data Deposition' section) and the respective accession numbers provided in the Data Availability Section?	Yes	Data Availability Section
Were **human clinical and genomic datasets** deposited in a public access-controlled repository in accordance to ethical obligations to the patients and to the applicable consent agreement?	Not Applicable	
Are **computational models** that are central and integral to a study available without restrictions in a machine-readable form? Were the relevant accession numbers or links provided?	Yes	Materials and Methods, Data Availability Section
If publicly available data were reused, provide the respective **data citations in the reference list**.	Yes	Figures, References
